# Endocrine therapy use and cardiovascular risk in postmenopausal breast cancer survivors

**DOI:** 10.1136/heartjnl-2020-317510

**Published:** 2020-11-11

**Authors:** Anthony A Matthews, Sharon Peacock Hinton, Susannah Stanway, Alexander Richard Lyon, Liam Smeeth, Jennifer L Lund, Krishnan Bhaskaran

**Affiliations:** 1 Institute of Environmental Medicine, Karolinska Institute, Stockholm, Sweden; 2 Department of Epidemiology, University of North Carolina at Chapel Hill, Chapel Hill, North Carolina, USA; 3 Department of Epidemiology and Population Health, London School of Hygiene & Tropical Medicine, London, UK; 4 Departmnet of Medicine, The Royal Marsden NHS Foundation Trust, London, UK; 5 National Heart and Lung Institute, Imperial College, London, UK; 6 Royal Brompton and Harefield NHS Trust, London, UK; 7 Lineberger Comprehensive Cancer Center, University of North Carolina at Chapel Hill, Chapel Hill, North Carolina, USA

**Keywords:** epidemiology, cardiac risk factors and prevention

## Abstract

**Objective:**

Examine the effect of tamoxifen and aromatase inhibitors (AIs) on the risk of 12 clinically relevant cardiovascular outcomes in postmenopausal female breast cancer survivors.

**Methods:**

We carried out two prospective cohort studies among postmenopausal women with breast cancer in UK primary care and hospital data (2002–2016) and US Surveillance, Epidemiology and End Results-Medicare data (2008–2013). Using Cox adjusted proportional hazards models, we compared cardiovascular risks between AI and tamoxifen users; and in the USA, between users of both drug classes and women receiving no endocrine therapy.

**Results:**

10 005 (UK) and 22 027 (USA) women with postmenopausal breast cancer were included. In both countries, there were higher coronary artery disease risks in AI compared with tamoxifen users (UK age-standardised incidence rate: 10.17 vs 7.51 per 1000 person-years, HR: 1.29, 95% CI 0.94 to 1.76; US age-standardised incidence rate: 36.82 vs 26.02 per 1000 person-years, HR: 1.29, 95% C I1.06 to 1.55). However, comparisons with those receiving no endocrine therapy (US data) showed no higher risk for either drug class and a lower risk in tamoxifen users (age-standardised incidence rate tamoxifen vs unexposed: 26.02 vs 35.19 per 1000 person-years, HR: 0.74, 95% 0.60 to 0.92; age-standardised incidence rate AI vs unexposed: 36.82 vs 35.19, HR: 0.96, 95% CI 0.83 to 1.10). Similar patterns were seen for other cardiovascular outcomes (arrhythmia, heart failure and valvular heart disease). As expected, there was more venous thromboembolism in tamoxifen compared with both AI users and those unexposed.

**Conclusions:**

Higher risks of several cardiovascular outcomes among AI compared with tamoxifen users appeared to be driven by protective effects of tamoxifen, rather than cardiotoxic effects of AIs.

## Background

Oestrogen or progesterone receptor positive breast cancer (ER/PR+) accounts for 83% of all breast cancer, and endocrine therapies are recommended to minimise risk of recurrence.^1 2^ Since 2006, aromatase inhibitors (AIs) have been recommended over tamoxifen for postmenopausal women due to greater efficacy.^3^ However, it has recently been suggested that AI users have a higher risk of subsequent cardiovascular disease (CVD) compared with tamoxifen users.^4^ It is in unclear whether this reflects cardiotoxicities of AIs or a protective effect of tamoxifen.

Tamoxifen inhibits the growth of breast tumours through competitive antagonism of oestrogen at its receptor. It is known that oestrogen agnostic effects of tamoxifen lowers total serum cholesterol by 10%–20% and low-density lipoprotein levels by 15%–22%, which could explain any protective effects of tamoxifen on the cardiovascular system.^5–8^ In contrast, the oestrogen-agonistic actions of tamoxifen result in a detrimental increase in thrombogenicity and increased risk of venous thrombosis and thromboembolism.^9^ AIs inhibit the conversion of the adrenal androgen substrate androstenedione to oestrogen in the breast tissue, reducing oestrogen production, and it has been hypothesised that the depletion of endogenous oestrogen production caused by AIs might increase the risk of CVD. A recent analysis of UK data observed higher risks of heart failure (HF) and possible higher risks of myocardial infarction (MI) and stroke in AI compared with tamoxifen users; it was suggested that the higher risks of cardiovascular events associated with AIs may therefore need to be taken account in treatment decisions.^10^ However, this study directly compared AIs and tamoxifen, and other evidence, summarised by a recent systematic review, has suggested that tamoxifen may have an underlying protective association with some CVD outcomes, making interpretation of the findings unclear.^4^

Using data on women with postmenopausal breast cancer from two large electronic medical record datasets, we therefore aimed to: (i) examine the consistency of estimated associations between endocrine therapy drug class (AI/tamoxifen) and CVD risk between UK and US cohorts and (2) compare CVD risks between users of both drug classes and ER/PR+ breast cancer patients unexposed to endocrine therapy to investigate the key question of whether differences between the two drug classes were driven by underlying harmful or protective associations.

## Methods

### Study design and data sources

We assembled two separate nationally representative cohorts of women with incident postmenopausal breast cancer using prospectively collected data from the UK and USA. In the UK, we used Clinical Practice Research Datalink primary care data (CPRD) and linked Hospital Episode Statistics (HES).^11^ In the USA, we used the Surveillance, Epidemiology, and End Results program (SEER) and Medicare linked database.^12^ Full data source details are in online supplemental appendix 1.

10.1136/heartjnl-2020-317510.supp1 Supplementary data

### Study populations

Differences in available data within the two cohorts are explained in online supplemental appendix 2.

#### UK cohort

We identified women with CPRD and HES data over 54 years (median age of the menopause in Europe^13^), with any incident breast cancer in CPRD (after at least 1 year of CPRD follow-up), who initiated AIs or tamoxifen in primary care after their diagnosis, from 1 January 2002 (date from which preliminary analysis showed that third-generation AIs came into widespread use) to 31 March 2016 (latest CPRD and HES linkage). Follow-up began at the latest of 1 year after breast cancer diagnosis or first AI or tamoxifen prescription (hereafter index date). Women were excluded if prior to their index date they: died, transferred out of CPRD, had any other cancer diagnosis or were diagnosed with the CVD event of interest (at any point prior to index date).

#### US cohort

We identified women over 65 years with incident ER/PR+ and stage 1–3 breast cancer and continuous Medicare Parts A, B and D enrolment (and no managed care coverage) for 12 months prior to the month of cancer diagnosis from 1 January 2008 (Medicare Part D data are available from 2007) and 31 December 2013 (last capture of cancer cases in SEER). Women with an endocrine therapy prescription prior to their breast cancer diagnosis were excluded. Follow-up began 1 year after the date of breast cancer (hereafter index date). Women were excluded if prior to their index date they: died, discontinued from Medicare Parts A, B or D, had any other cancer diagnosis or were diagnosed with the CVD event of interest (within a 3-year look back period to ensure likelihood of capturing a prior event was not dependent on age as Medicare follow-up starts at 65 years).

### Exposure, outcomes and covariates

Tamoxifen and AI exposures were identified using prescription codes in the UK (available at https://doi.org/10.17037/DATA.177), and National Drug Codes and Healthcare Common Procedural Coding System (HCPCS) procedure codes in the USA (online supplemental appendix 3). Primary exposure was ever use of tamoxifen, ever use of AI or ever use of both drugs, with ever use defined as at least one prescription (UK) or fill (USA) of the endocrine therapy medication; the US study additionally included no exposure to any endocrine therapy (which did not exist in the UK study, because receipt of endocrine therapy was an inclusion criterion). Exposure was time-updated to indicate a woman had been exposed to both drugs if they switched endocrine therapies during follow-up. The baseline exposure group was classified as ever exposure to tamoxifen for the ever AI versus tamoxifen analyses in both the UK and USA. The baseline exposure group was also changed to no exposure to any endocrine therapy for the ever AI/tamoxifen versus unexposed analyses that was only possible in the USA. Visualisations of exposure categorisations are shown in online supplemental appendix 4. The main CVD outcomes were: coronary artery disease (angina, MI, revascularisation procedures and sudden cardiac arrest); peripheral vascular disease; stroke; arrhythmia; HF (including cardiomyopathy); pericarditis; valvular heart disease (VHD); and venous thromboembolism (VTE) (deep vein thrombosis (DVT) and pulmonary embolism). Composite CVD outcomes and individual components of the composite outcomes were analysed separately. Events were identified through clinical diagnoses using NHS Read codes in the CPRD and International Classification of Disease (ICD), 10th edition codes in HES in the UK (available at https://doi.org/10.17037/DATA.177) and ICD-9 and HCPCS codes in the US study (online supplemental appendix 5).

In both studies, we adjusted for age, cardiovascular history and risk factors, use of cardioprotective medications, other comorbidities, time since index date and calendar year. In the UK, we were additionally able to adjust for smoking, alcohol, body mass index and index of multiple deprivation (ie, socioeconomic status); in the US study, we were additionally able to adjust for race, region and use of anticancer therapies (anthracyclines, taxanes, trastuzumab and other systemic treatments). A full comparison of the covariates considered in the UK and USA is in online supplemental appendix 6. Algorithms to define confounders in the UK are in online supplemental appendix 7, and code lists used for variable definitions are at https://doi.org/10.17037/DATA.177. Codes used to identify prescriptions in the US are in online supplemental appendix 8.

### Statistical analysis

Observation began at index date and ended at the earliest of: a CVD event of interest; diagnosis of another (non-breast) cancer; death; and transfer out of CPRD/end of Medicare Parts A, B or D enrolment. Observation could also end at the end of the study period, which was 31 March 2016 in UK and 31 December 2014 in USA.

Separate analyses were conducted in UK and USA and for each CVD outcome. Distributions of characteristics at index date were described. Number of events and crude incident rates of each outcome of interest by exposure were calculated. Cox proportional hazards models with an age timescale were fitted for each outcome of interest to obtain unadjusted HRs and 95% CIs for the association between endocrine therapy use and outcome; those with a diagnosis of the specific outcome of interest before the index date were excluded from the analysis for that outcome. All covariates were then added to obtain fully adjusted HRs. We then fitted interactions, with prespecified variables considered clinically important to investigate effect modification by current age (54–69 and 70+ years in the UK; 66–84 and 85+ years in the USA); time since index date (0–1 years, 1–3 years and 3+ years); and history of any CVD prior to index date (other than the CVD outcome of interest) for the coronary artery disease (composite), arrhythmia, stroke, pericarditis (USA only), HF, VHD and VTE (composite) outcomes (interactions were not investigated for other outcomes due to limited power). We statistically tested for interactions using likelihood ratio tests. Women with missing data (8.7% in the UK, and 5.1% in the USA) were excluded from all analyses (complete case analysis), which is valid in a regression context if missingness is conditionally independent of the outcome.^14^ Unfortunately, this is an untestable assumption (as it includes conditioning on the missing values themselves), but this assumption is more plausible than the missing at random assumption required for multiple imputation in the UK data; in the US data, it is possible that missingness was either at random and conditionally independent of the outcome, but we used a complete case analysis in order to have a consistent approach across the UK and US studies. We also calculated age-standardised incidence rates for each outcome by fitting a Poisson regression model with exposure and age, setting all individuals to each level of exposure separately and predicting the number of events, then dividing by the total person-years.

### Sensitivity analyses

The main analyses were repeated with the UK and US study populations and covariates modified to be as similar as possible (details in online supplemental appendix 9) A post hoc quantitative bias analysis explored potential unmeasured confounding in the large estimated protective effect of tamoxifen use on the risk of MI (online supplemental appendix 10).^15^ Finally, the primary analysis in the US data was additionally adjusted adjuvant radiotherapy to assess any additional confounding of the effect of endocrine therapy on the risk of CVD.

## Results

**Figure 1:**
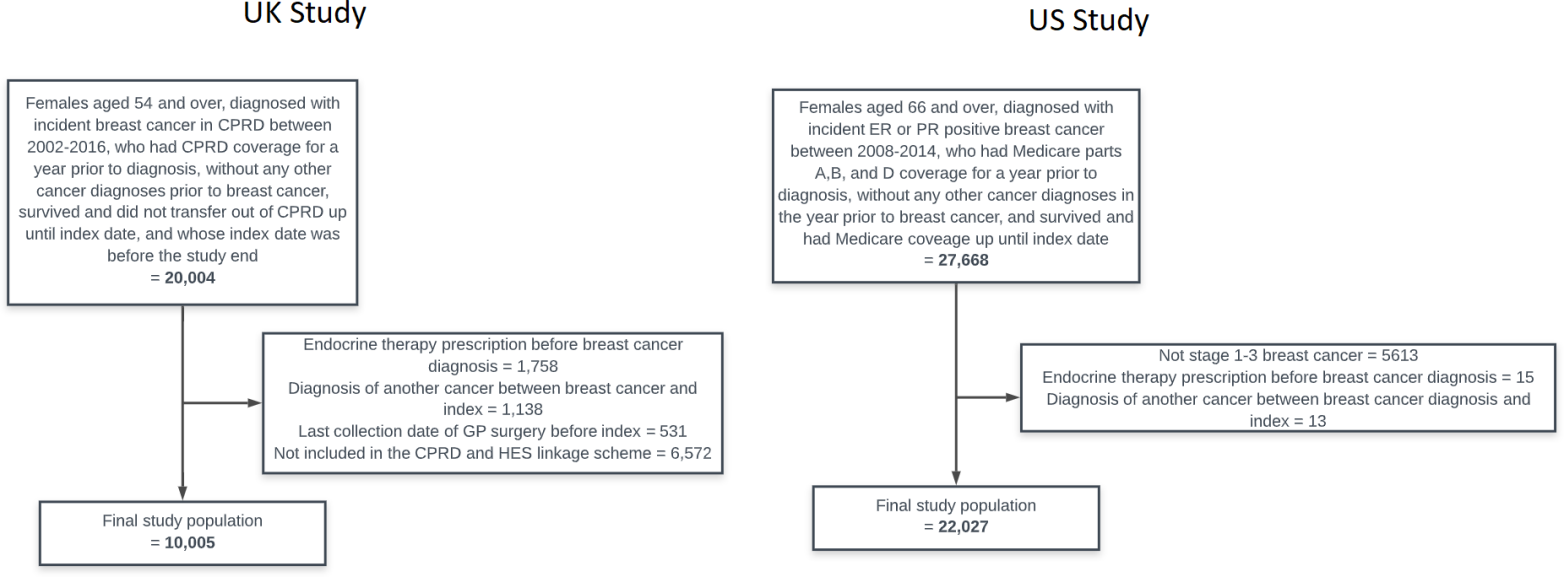
Flow diagrams of study populations in the UK and USA. CPRD, Clinical Practice Research Datalink; GP, general practitioner; HES, Hospital Episode Statistics; ER, oestrogen; PR, progesterone.

The UK study included 10 005 women, with 4716 (47%) initially prescribed tamoxifen and 5289 (53%) initially prescribed an AI (table 1, flow diagram in figure 1); the median person-years of follow-up in each exposure group was: ever tamoxifen: 2.3 years (IQR: 1.2–5.4 years), ever AI: 2.2 years (IQR: 0.9–4.3 years) and ever both: 3.5 years (IQR: 1.5–6.3 years). The US study included 22 027 women, with 4667 (22%), 2286 (10%) and 15 074 (68%) initially filling no endocrine therapy, tamoxifen and an AI, respectively (table 2, flow diagram in figure 1); the median person-years of follow-up in each exposure group were as follows: unexposed: 1.6 years (IQR: 0.6–3.3 years), tamoxifen 2.2 years (IQR: 1.0–3.8 years), AI: 2.0 years (IQR: 0.9–3.6 years) and both: 1.9 years (IQR: 0.8–3.5 years).

**Table 1:** Characteristics of study population based on their initial exposure in the UK

	Tamoxifen	AI	Total
**N**	4716 (100)	5289 (100)	10 005 (100)
**Age (years)**			
54–59	911 (19.3)	716 (13.5)	1627 (16.3)
60–69	1850 (39.2)	1972 (37.3)	3822 (38.2)
70+	1955 (41.5)	2601 (49.2)	4556 (45.5)
Median (IQR)	68 (62–76)	70 (63–79)	69 (62–78)
**Year of breast cancer**			
2002–2005	2267 (48.1)	670 (12.7)	2937 (29.4)
2006–2009	1523 (32.3)	1846 (34.9)	3369 (33.7)
2010–2013	823 (17.5)	2248 (42.5)	3071 (30.7)
2014–2015	103 (2.2)	525 (9.9)	628 (6.3)
**BMI (kg/m^2^)**			
<18	59 (1.3)	63 (1.2)	122 (1.2)
18–24	1693 (35.9)	1619 (30.6)	3312 (33.1)
25–29	1549 (32.8)	1801 (34.1)	3350 (33.5)
30–34	800 (17)	979 (18.5)	1779 (17.8)
≥35	345 (7.3)	548 (10.4)	893 (8.9)
*Missing*	270 (5.7)	279 (5.3)	549 (5.5)
Median (IQR)	26 (23–30)	27 (24–31)	27 (24–31)
**Smoking status**			
Never smoker	2423 (51.4)	2517 (47.6)	4940 (49.4)
Current smoker	503 (10.7)	482 (9.1)	985 (9.8)
Ex-smoker	1761 (37.3)	2268 (42.9)	4029 (40.3)
*Missing*	29 (.6)	22 (.4)	51 (.5)
**Alcohol use**			
Non-drinker	618 (13.1)	613 (11.6)	1231 (12.3)
Current	3320 (70.4)	3628 (68.6)	6948 (69.4)
Ex-drinker	480 (10.2)	715 (13.5)	1195 (11.9)
*Missing*	298 (6.3)	333 (6.3)	631 (6.3)
**Systolic BP**			
Low/ideal	530 (11.2)	599 (11.3)	1129 (11.3)
Prehigh	1862 (39.5)	2327 (44)	4189 (41.9)
High	2314 (49.1)	2355 (44.5)	4669 (46.7)
*Missing*	10 (.2)	8 (.2)	18 (.2)
**Diastolic BP**			
Low/ideal	2130 (45.2)	2650 (50.1)	4780 (47.8)
Prehigh	1988 (42.2)	2058 (38.9)	4046 (40.4)
High	588 (12.5)	573 (10.8)	1161 (11.6)
*Missing*	10 (.2)	8 (.2)	18 (.2)
**Index of Multiple deprivation category**			
1	870 (18.4)	962 (18.2)	1832 (18.3)
2	943 (20)	1214 (23)	2157 (21.6)
3	925 (19.6)	1054 (19.9)	1979 (19.8)
4	1052 (22.3)	936 (17.7)	1988 (19.9)
5	926 (19.6)	1122 (21.2)	2048 (20.5)
*Missing*	0 (0)	1 (0)	1 (0)
**CVD-related treatment before index**			
Statins	1100 (23.3)	1903 (36)	3003 (30)
ACEi	1195 (25.3)	1823 (34.5)	3018 (30.2)
CCB	1195 (25.3)	1764 (33.4)	2959 (29.6)
ARB	493 (10.5)	795 (15)	1288 (12.9)
Antiplatelets	1132 (24)	1639 (31)	2771 (27.7)
**Comorbidities before index**			
RA	138 (2.9)	137 (2.6)	275 (2.7)
Diabetes	462 (9.8)	728 (13.8)	1190 (11.9)
CKD	867 (18.4)	1090 (20.6)	1957 (19.6)
**CVD before index**			
Non-venous CVD	1031 (21.9)	1636 (30.9)	2667 (26.7)
VTE before index	144 (3.1)	324 (6.1)	468 (4.7)

ACEi, Angiotensin-converting-enzyme inhibitors; AI, aromatase inhibitor; ARB, angiotensin II receptor blocker; BP, blood pressure; CCB, calcium channel blocker; CKD, Chronic kidney disease; CVD, cardiovascular disease; RA, Rhematoid arthritis; VTE, venous thromboembolism.

**Table 2:** Characteristics of study population based on their initial exposure in the USA

	Unexposed	Tamoxifen	AI	Total
**N**	4667 (100)	2286 (100)	15 074 (100)	22 027 (100)
**Age at index date (years)**				
66–74	1538 (33)	897 (39.2)	7505 (49.8)	9940 (45.1)
75–84	1937 (41.5)	994 (43.5)	5894 (39.1)	8825 (40.1)
85+	1192 (25.5)	395 (17.3)	1675 (11.1)	3262 (14.8)
Median (IQR)	79 (73–85)	77 (72–83)	75 (71–81)	76 (71–82)
Ethnicity*				
White	4002 (85.8)	2028 (88.7)	12 782 (84.8)	18 812 (85.4)
Black	360 (7.7)	102 (4.5)	1099 (7.3)	1561 (7.1)
Other	93 (2)	47 (2.1)	335 (2.2)	475 (2.2)
Asian	123 (2.6)	68 (3)	498 (3.3)	689 (3.1)
Hispanic	–	–	–	397 (1.8)
Native American	–	–	–	52 (.2)
*Missing*	–	–	–	41 (.2)
**SEER region**				
North East	760 (16.3)	283 (12.4)	3320 (22)	4363 (19.8)
South	1023 (21.9)	605 (26.5)	3778 (25.1)	5406 (24.5)
North Central	695 (14.9)	448 (19.6)	1761 (11.7)	2904 (13.2)
West	2157 (46.2)	939 (41.1)	6130 (40.7)	9226 (41.9)
*Missing*	32 (.7)	11 (.5)	85 (.6)	128 (.6)
**Year of breast cancer**				
2008–2009	1678 (36)	1231 (53.8)	6609 (43.8)	10 247 (46.5)
2010–2011	1473 (31.6)	701 (30.7)	5019 (33.3)	7193 (32.7)
2012–2013	1516 (32.5)	722 (31.6)	5840 (38.7)	8078 (36.7)
**Stage of breast cancer**				
Stage I	3034 (65)	1486 (65)	8379 (55.6)	12 899 (58.6)
Stage II	1275 (27.3)	660 (28.9)	5267 (34.9)	7202 (32.7)
Stage III	358 (7.7)	140 (6.1)	1428 (9.5)	1926 (8.7)
**Grade of breast cancer**				
1	1522 (32.6)	765 (33.5)	4273 (28.3)	6560 (29.8)
2	2071 (44.4)	1109 (48.5)	7350 (48.8)	10 530 (47.8)
3	853 (18.3)	324 (14.2)	2810 (18.6)	3987 (18.1)
*Missing*	221 (4.7)	88 (3.8)	641 (4.3)	950 (4.3)
**Cancer treatments**				
Taxane	570 (12.2)	162 (7.1)	2415 (16)	3147 (14.3)
Anthracyclines	259 (5.5)	68 (3)	820 (5.4)	1147 (5.2)
Trastuzumab	226 (4.8)	39 (1.7)	687 (4.6)	952 (4.3)
Other treatment	753 (16.1)	244 (10.7)	2992 (19.8)	3989 (18.1)
**Comorbidities**				
RA	185 (4)	103 (4.5)	547 (3.6)	835 (3.8)
CKD	383 (8.2)	155 (6.8)	1113 (7.4)	1651 (7.5)
Hypertension	3426 (73.4)	1612 (70.5)	11 113 (73.7)	16 151 (73.3)
Diabetes	1313 (28.1)	598 (26.2)	4545 (30.2)	6456 (29.3)
**CVD-related treatment before index**				
Statins	1778 (38.1)	948 (41.5)	6988 (46.4)	9714 (44.1)
Hypertensives	169 (3.6)	83 (3.6)	577 (3.8)	829 (3.8)
ACEi	962 (20.6)	477 (20.9)	3251 (21.6)	4690 (21.3)
CCB	850 (18.2)	364 (15.9)	2696 (17.9)	3910 (17.8)
ARB	593 (12.7)	255 (11.2)	2063 (13.7)	2911 (13.2)
**Past CVD**				
Non-venous CVD	2989 (64)	1281 (56)	8896 (59)	13 166 (59.8)
VTE	162 (3.5)	31 (1.4)	385 (2.6)	578 (2.6)

*Cell numbers within ethnicity suppressed due to some cells containing numbers <11.

ACEi, Angiotensin-converting-enzyme inhibitors; AI, aromatase inhibitor; ARB, angiotensin II receptor blocker; CCB, calcium channel blocker; CKD, Chronic kidney disease; CVD, cardiovascular disease; RA, Rheumatoid arthritis; SEER, Surveillance, Epidemiology, and End Results; VTE, venous thromboembolism.

### Ever AI versus tamoxifen use

**Figure 2:**
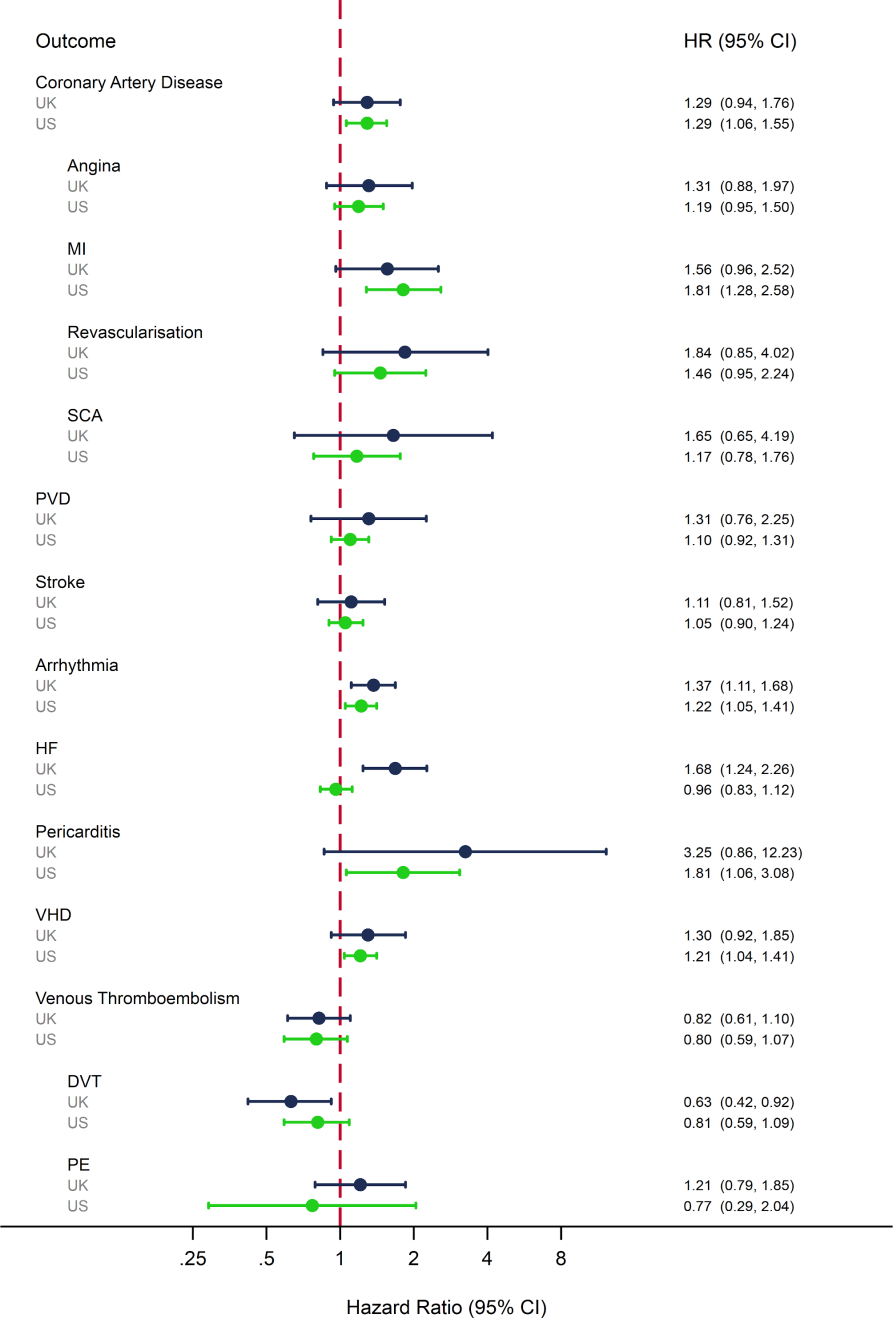
Adjusted HRs for the association between ever AI use compared with ever tamoxifen use and the risk of a range of clinical CVD outcomes in the UK and USA. *UK results adjusted for the following covariates at baseline: for age (54–59, 60–69 and 70+ years); smoking status (non-smoker, current smoker and ex-smoker); BMI (underweight/healthy weight, overweight and obese); alcohol status (non-drinker, current drinker and ex-drinker); IMD score (levels 1–5 based on GP level IMD data); use of statins; use of ACE inhibitors; use of calcium channel blockers; use of angiotensin II receptor blockers; diabetes; chronic kidney disease; rheumatoid arthritis; systolic blood pressure (low/normal, prehigh and high); diastolic blood pressure (low/normal, prehigh and high); history of non-venous CVD year of breast cancer diagnosis; time since index (0–1 years, 1–3 years, 3–5 years and 5+ years); and current year. US results adjusted for year of breast cancer diagnosis (2007–2013); age at index date (66–74, 75–84 and 85+ years); race (white, black Asian, Hispanic, Native American and other); SEER region (North East, South, North Central and West); breast cancer stage (1–3); breast cancer grade (1–3); time since index date (0–1years, 1–3 years, 3–5 years and 5+ years); current calendar year; use of taxanes, anthracycline, trastuzumab, other systemic cancer treatments, statins, antihypertensive drugs, ACE inhibitors, calcium channel blockers, angiotensin receptor blockers; diagnosis of rheumatoid arthritis, chronic kidney disease, hypertension, diabetes, VTE and non-venous CVD. **Numbers of events for each outcome in the AI and tamoxifen groups are shown in online supplemental appendices 11 and 12. AI, aromatase inhibitor; BMI, body mass index; CVD, cardiovascular disease; DVT, deep vein thrombosis; HF, heart failure; MI, myocardial infarction; PE, pulmonary embolism; PVD, peripheral vascular disease; SCA, sudden cardiac arrest; SEER, Surveillance, Epidemiology, and End Results; VHD, valvular heart disease; VTE, venous thromboembolism.

In both the UK and USA, there was a higher observed rate of most CVDs, excluding VTE outcomes, among those ever exposed to an AI compared with tamoxifen (online supplemental appendices 11 and 12). In adjusted analyses, there was evidence of a higher risk of HF in AI compared with tamoxifen users in the UK (HR: 1.68, 95% CI 1.24 to 2.26; figure 2), which was not replicated in the USA (HR: 0.96, 95% CI 0.83 to 1.12). Other adjusted HRs were consistent between the two settings; there was evidence in one or both settings that AI users compared with tamoxifen users had higher risk of coronary artery disease, MI, arrhythmia, pericarditis and VHD (HRs ranged from 1.29 to 3.25 in the UK, and 1.21 to 1.81 in the USA; figure 2) and lower risk of DVT (UK HR: 0.63, 95% 0.43 to 0.93; US HR: 0.81, 95% CI 0.50 to 1.09).

### Ever AI/tamoxifen use versus unexposed

**Figure 3:**
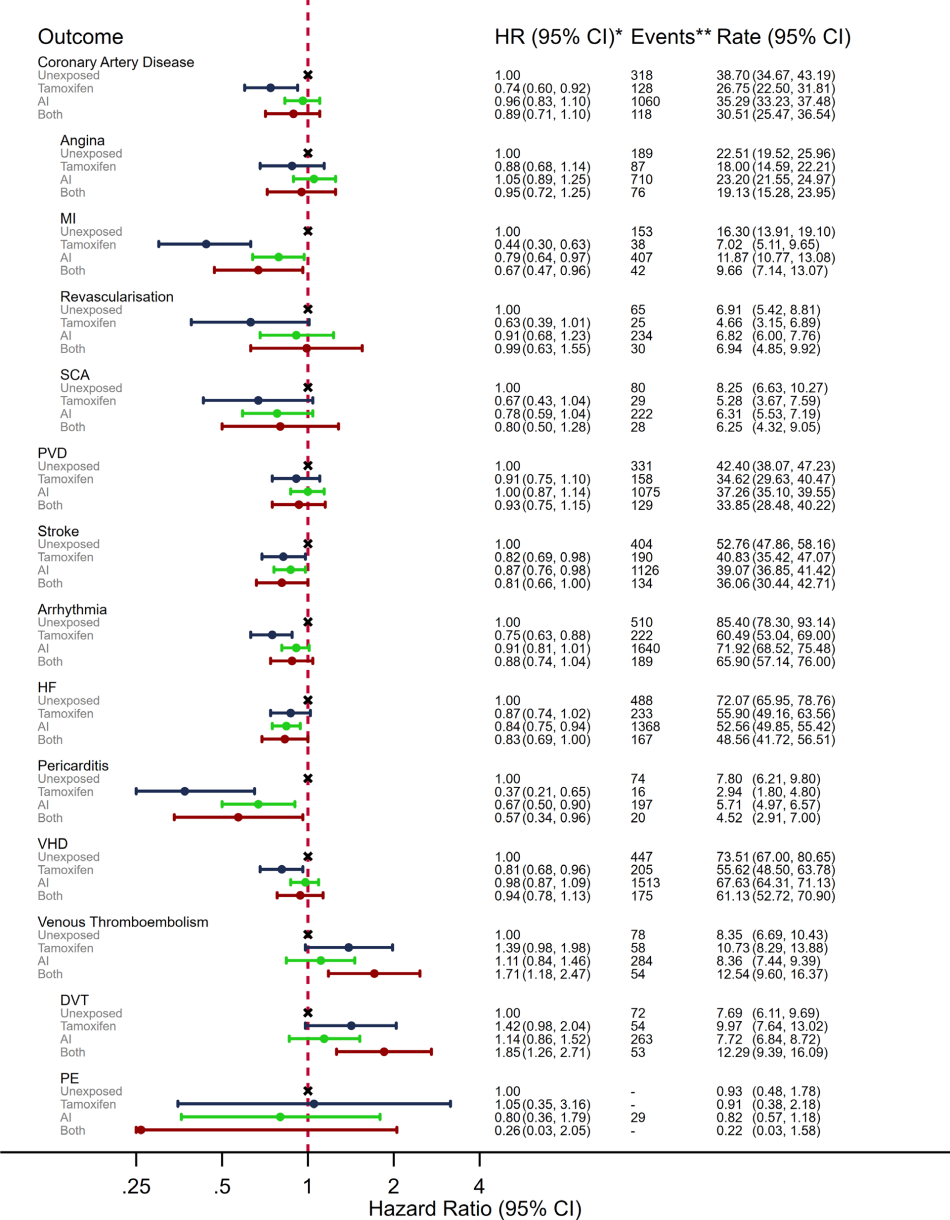
Adjusted HRs, events and crude rate per 1000 person-years for the association between ever exposure to endocrine therapy and a range of clinical CVD outcomes in the USA. *Adjusted for year of breast cancer diagnosis (2007–2013); age at index date (66–74, 75–84 and 85+ years); race (white, black Asian, Hispanic, Native American and other); SEER region (North East, South, North Central and West); breast cancer stage (1–3); breast cancer grade (1–3); time since index date (0–1 year, 1–3 years, 3–5 years and 5+ years); current calendar year; use of taxanes, anthracyclines, trastuzumab, other systemic cancer treatments, statins, antihypertensive drugs, ACE inhibitors, calcium channel blockers, angiotensin receptor blockers; and diagnosis of rheumatoid arthritis, chronic kidney disease, hypertension, diabetes, VTE and non-venous CVD. **Events and follow-up suppressed if number of events ≤11. CVD, cardiovascular disease; DVT, deep vein thrombosis; HF, heart failure; MI, myocardial infarction; PE, pulmonary embolism; PVD, peripheral vascular disease; SCA, sudden cardiac arrest; SEER, Surveillance, Epidemiology, and End Results; VHD, valvular heart disease; VTE, venous thromboembolism.

The US study included women without exposure to either endocrine therapy; observed rates of most CVDs, excluding VTE, were lower in both the tamoxifen and AI groups compared with the unexposed group, while VTE outcome rates were higher in the endocrine therapy groups (Appendix 12). The patterns were similar in adjusted analyses (figure 3); there was evidence that tamoxifen users had lower risks than unexposed women for coronary artery disease, MI, stroke, arrhythmia, pericarditis and VHD (HRs ranged from 0.37 to 0.87); HR point estimates for AI users versus unexposed were also in the protective direction for all non-VTE CVD outcomes, but in mostly closer to the null than for tamoxifen, and with confidence intervals including no association. There was weak evidence of higher risk VTE in tamoxifen users compared with the unexposed (HR: 1.39, 95% CI 0.98 to 1.98) but little evidence of a difference for AI users versus unexposed (HR: 1.14, 95% Ci 0.86 to 1.52).

### Effect modification

There was no strong evidence of effect modification by age, time since index date or prior CVD in the UK or USA (online supplemental appendices 13 and 14), though there were few events within strata, limiting precision. There was a suggestion in UK data that the raised risk of coronary artery disease in AI compared with tamoxifen users diminished over time (p=0.02) but no corresponding evidence in US data.

### Sensitivity analyses

Following modification of study populations, methodology and covariates in the UK and USA to make them as similar as possible, the risk of all CVDs associated with ever AI compared with tamoxifen use were generally in the same direction (online supplemental appendix 15). However, the discrepant results for HF between the two cohorts persisted. Quantitative bias analyses suggest that unmeasured confounding was unlikely to fully explain the large HR for the effect of ever tamoxifen use on MI risk (online supplemental appendix 16). Effect estimates in US data after additional adjustment for adjuvant radiotherapy were similar to primary results (online supplemental appendix 17).

## Discussion

Our results suggest that higher observed risks of cardiovascular outcomes including coronary artery disease, MI, arrhythmia, HF, pericarditis and VHD in AI users compared with tamoxifen users are driven by protective associations of tamoxifen with CVD outcomes, rather than any cardiotoxic effects of AIs. These two population-based cohort studies using UK and US data are the first to apply similar methodology to two large populations to assess the effects of endocrine therapies on a range of CVD outcomes in postmenopausal women with breast cancer. Both countries’ results suggested a higher risk of several CVD outcomes in AI compared with tamoxifen users. However, when compared with patients receiving no endocrine therapy, there was no raised risk of any CVD outcome in users of either drug class, other than VTE in tamoxifen users. Furthermore, tamoxifen users had lower risks of coronary artery disease, MI, stroke, arrhythmia, pericarditis and VHD than unexposed women. This protective effect of tamoxifen might be explained by the drug’s effect on lipid levels; previous studies have found tamoxifen to reduce cholesterol.^5 16^

### Comparison with other studies

A recent meta-analysis of trials reported an increased risk of CVD, excluding VTE, in tamoxifen compared with AI users (RR: 1.18, 95% CI 1.05 to 1.33),^17^ with results suggestive of a cardioprotective effect of tamoxifen, consistent with the results of this study.^10^

Most (5/6) previous studies directly comparing the risk of MI in AI and tamoxifen users have reported a higher risk in AI users, similar to our results (RRs ranged from 0.99 to 2.02).^10 18–22^ Two previous trials and five observational studies have compared MI risk in tamoxifen users with non-use/placebo, with 4/7 studies finding a reduced risk in tamoxifen users (RRs ranged from 0.20 to 0.83)^23–29^; two analogous studies of AI use versus placebo or non-use found no association.^28 30^ Seven studies (five trials and two observational) directly compared the risk of stroke in AI and tamoxifen users, but effects in both directions have been reported.^20 22 31–34^ Three out of five studies (one trial and four observational) comparing tamoxifen use with non-use/placebo found a protective association with stroke, as in our study (RRs ranged from 0.52 to 0.81).^23 26–28 35^ In the present analysis, we found AI users to be at lower risk of stroke compared with unexposed women; one previous study found a similar association but a second reported the opposite.^28 30^ Three previous studies (one trial and two observational) have reported results for the comparison between AI and tamoxifen use on the risk of HF,^10 31 34^ for which we reported discrepant results between the UK and USA. Two reported a higher risk in AI users and the other reported no association. The discrepant results in our study could be due to residual confounding by variables not available in both datasets (cancer therapies in the UK and lifestyle factors in the USA), or the nature of the data sources (routinely collected records in the UK and claims in the USA).

Six previous trials compared the risk of VTE in AI and tamoxifen users, with five reporting a lower risk in AI compared with tamoxifen users (RR ranged from 1.25 to 0.61), as reported in both UK and US data in this study.^18 20 22 31 36 37^ Six out of eight studies (five trials and three observational) also suggested a higher risk in tamoxifen users (RRs ranged from 1.64 to 7.10), which is in the same direction, but larger than the effect in US data here.

### Strengths and limitations

A major strength of this study was the use of two large data sources with complementary strengths and different limitations. We could look for consistency between countries and conduct different comparisons, notably between classes and with unexposed patients. We were also able to assess the relationships between endocrine therapy treatments and a wide range of CVD outcomes, rather than the composite and individual CVD outcomes in previous studies. We were able to account for several important confounders such as potentially cardiotoxic treatments (in US data) and lifestyle factors (in UK data), although no confounders materially changed the crude effect estimate when adjusted for individually in either the UK or USA (online supplemental appendices 18 and 19). As the CPRD broadly represents the UK population, and SEER-Medicare includes a large, diverse population of older women diagnosed with breast cancer, results are generalisable to women diagnosed with ER/PR+ breast cancer in both the UK, USA and other developed populations due to the homogenous indication of endocrine therapy worldwide.

In the UK, ER/PR status was not available, but it is likely that breast cancers were ER/PR+ as such a diagnosis is a prerequisite of being prescribed endocrine therapies. Detail on cancer stage and administered therapies were also not available in the UK; although the cancer diagnosis is fed back from specialists to general practitioners, further detail is typically not recorded by the general practitioner and hence not available in the CPRD. HES also does not include these data; it is not a clinical care database and is rather derived from administrative data within the National Health Service. Furthermore, CPRD captures prescriptions at the point of issue, but we do not know if prescriptions were filled, which could lead to potential misclassification of exposure. However, descriptive analyses using a 3-month grace period to define a continuous prescription indicated that 94% of women continued to be prescribed within 1 year of starting, 85% within 3 years and 74% within 5 years.

In the USA, the proportion of non-initiators of endocrine therapy (21%) was similar to the proportion of non-initiators reported in a previous study.^17^ Reasons for non-initiation may include frailty, poor CVD preventative care and high BMI, introducing possible residual confounding. Although quantitative bias analyses suggest it is unlikely that residual confounding explains all the large observed protective associations between tamoxifen use and several CVD outcomes, these associations may be exaggerated, and the smaller observed associations between AI use and outcomes could be driven by such confounding. Comparisons with non-user groups are often more subject to confounding than comparisons with groups using similar medications (ie, AI vs tamoxifen).^38^ Caution should be taken in the interpretation of the comparisons with non-users, and these results warrant further investigation. Furthermore, the US analyses did not include those in a managed care programme (Medicare Advantage) as these plans do not report claim-level information to Medicare, so these data cannot be used for research. Between 2007 and 2013, this population ranged from approximately 19%–28% of the total Medicare population, and it is understood that they have lower healthcare utilisation and higher quality of care.^39–41^

## Conclusion

Among postmenopausal women diagnosed with ER/PR+ breast cancer, we found convincing evidence of a higher risk of several CVDs in AI compared with tamoxifen users. However, our results indicated no excess cardiotoxicities of either drug class, other than the known raised risk of VTE with tamoxifen use. There was no evidence of raised risk of any specific CVD with AI use, including in stratified results restricted to those with other prior CVDs, suggesting that a history of CVD is unlikely to be an important contraindication for prescription of an AI. Our results contribute to the evidence base regarding the risk–benefit balance of endocrine therapies with respect to cancer survival and cardiovascular outcomes and ultimately can help identify subgroups of postmenopausal women who are likely to benefit from tamoxifen over AIs.

**Key messages**

**What is already known on this subject?**

- Women with oestrogen or progesterone receptor positive breast cancer typically receive endocrine therapies, either tamoxifen or aromatase inhibitors (AIs), to reduce cancer recurrence risk. Recent studies have suggested raised cardiovascular risks in AI compared with tamoxifen users, but it is unclear whether this reflects cardiotoxicities of AIs or a protective effect of tamoxifen.

**What might this study add?**

- Among postmenopausal women with breast cancer, we found a higher risk of several cardiovascular diseases in AI compared with tamoxifen users across two countries, which appeared to be driven by protective effects of tamoxifen, rather than toxic effects of AIs. We also found the known higher venous thromboembolism risk in tamoxifen users.

**How might this impact on clinical practice?**

- Previous evidence could lead to reduced uptake of AIs among those at a high risk of cardiovascular disease. However, there is no current evidence to suggest that the cardiovascular benefits of tamoxifen outweigh the far superior effect that AIs have on breast cancer recurrence in this population, and there is no higher cardiovascular risk when prescribing AIs to these patients. These results should allay any concerns about postulated cardiotoxicities of AIs, and modifications to clinical practice could reduce the risk of patients being prescribed inappropriate endocrine therapies.

## References

[1] Howlader N , Altekruse SF , Li CI , et al . US incidence of breast cancer subtypes defined by joint hormone receptor and HER2 status. J Natl Cancer Inst 2014;106. 10.1093/jnci/dju055. [Epub ahead of print: 28 Apr 2014]. PMC4580552 24777111

[2] , Davies C , Godwin J , et al, Early Breast Cancer Trialists' Collaborative Group (EBCTCG) . Relevance of breast cancer hormone receptors and other factors to the efficacy of adjuvant tamoxifen: patient-level meta-analysis of randomised trials. Lancet 2011;378:771–84. 10.1016/S0140-6736(11)60993-8 21802721 PMC3163848

[3] Dowsett M , Cuzick J , Ingle J , et al . Meta-analysis of breast cancer outcomes in adjuvant trials of aromatase inhibitors versus tamoxifen. J Clin Oncol 2010;28:509–18. 10.1200/JCO.2009.23.1274 19949017

[4] Matthews A , Stanway S , Farmer RE , et al . Long term adjuvant endocrine therapy and risk of cardiovascular disease in female breast cancer survivors: systematic review. BMJ 2018;363:k3845. 10.1136/bmj.k3845 30297439 PMC6174332

[5] Dewar JA , Horobin JM , Preece PE , et al . Long term effects of tamoxifen on blood lipid values in breast cancer. BMJ 1992;305:225–6. 10.1136/bmj.305.6847.225 1392827 PMC1882673

[6] Esteva FJ , Hortobagyi GN . Comparative assessment of lipid effects of endocrine therapy for breast cancer: implications for cardiovascular disease prevention in postmenopausal women. Breast 2006;15:301–12. 10.1016/j.breast.2005.08.033 16230014

[7] Grey AB , Stapleton JP , Evans MC , et al . The effect of the anti-estrogen tamoxifen on cardiovascular risk factors in normal postmenopausal women. J Clin Endocrinol Metab 1995;80:3191–5. 10.1210/jcem.80.11.7593425 7593425

[8] Morales M , Santana N , Soria A , et al . Effects of tamoxifen on serum lipid and apolipoprotein levels in postmenopausal patients with breast cancer. Breast Cancer Res Treat 1996;40:265–70. 10.1007/BF01806815 8883969

[9] Saphner T , Tormey DC , Gray R . Venous and arterial thrombosis in patients who received adjuvant therapy for breast cancer. J Clin Oncol 1991;9:286–94. 10.1200/JCO.1991.9.2.286 1988575

[10] Khosrow-Khavar F , Filion KB , Bouganim N , et al . Aromatase inhibitors and the risk of cardiovascular outcomes in women with breast cancer: a population-based cohort study. Circulation 2020;141:549–59. 10.1161/CIRCULATIONAHA.119.044750 32065766

[11] Herrett E , Gallagher AM , Bhaskaran K , et al . Data resource profile: clinical practice research Datalink (CPRD). Int J Epidemiol 2015;44:827–36. 10.1093/ije/dyv098 26050254 PMC4521131

[12] Warren JL , Klabunde CN , Schrag D , et al . Overview of the SEER-Medicare data: content, research applications, and generalizability to the United States elderly population. Med Care 2002;40:IV-3-18. 10.1097/01.MLR.0000020942.47004.03 12187163

[13] Dratva J , Gómez Real F , Schindler C , et al . Is age at menopause increasing across Europe? Results on age at menopause and determinants from two population-based studies. Menopause 2009;16:385–94. 10.1097/gme.0b013e31818aefef 19034049

[14] White IR , Carlin JB . Bias and efficiency of multiple imputation compared with complete-case analysis for missing covariate values. Stat Med 2010;29:2920–31. 10.1002/sim.3944 20842622

[15] Lash TF , Fink, AK MP . Applying quantitative bias analysis to epidemiologic data. Springer, 2009.

[16] Love RR , Wiebe DA , Feyzi JM , et al . Effects of tamoxifen on cardiovascular risk factors in postmenopausal women after 5 years of treatment. J Natl Cancer Inst 1994;86:1534–9. 10.1093/jnci/86.20.1534 7932809

[17] Khosrow-Khavar F , Filion KB , Al-Qurashi S , et al . Cardiotoxicity of aromatase inhibitors and tamoxifen in postmenopausal women with breast cancer: a systematic review and meta-analysis of randomized controlled trials. Ann Oncol 2017;28:487–96. 10.1093/annonc/mdw673 27998966 PMC5834146

[18] Jakesz R , Jonat W , Gnant M , et al . Switching of postmenopausal women with endocrine-responsive early breast cancer to anastrozole after 2 years' adjuvant tamoxifen: combined results of ABCSG trial 8 and ARNO 95 trial. Lancet 2005;366:455–62. 10.1016/S0140-6736(05)67059-6 16084253

[19] Coombes RC , Kilburn LS , Snowdon CF , et al . Survival and safety of exemestane versus tamoxifen after 2-3 years' tamoxifen treatment (intergroup Exemestane study): a randomised controlled trial. Lancet 2007;369:559–70. 10.1016/S0140-6736(07)60200-1 17307102

[20] Pagani O , Regan MM , Walley BA , et al . Adjuvant exemestane with ovarian suppression in premenopausal breast cancer. N Engl J Med 2014;371:107–18. 10.1056/NEJMoa1404037 24881463 PMC4175521

[21] Abdel-Qadir H , Amir E , Fischer HD , et al . The risk of myocardial infarction with aromatase inhibitors relative to tamoxifen in post-menopausal women with early stage breast cancer. Eur J Cancer 2016;68:11–21. 10.1016/j.ejca.2016.08.022 27693889

[22] Arimidex, Tamoxifen, Alone or in Combination (ATAC) Trialists' Group, Forbes JF , Cuzick J , et al . Effect of anastrozole and tamoxifen as adjuvant treatment for early-stage breast cancer: 100-month analysis of the ATAC trial. Lancet Oncol 2008;9:45–53. 10.1016/S1470-2045(07)70385-6 18083636

[23] McDonald CC , Alexander FE , Whyte BW , et al . Cardiac and vascular morbidity in women receiving adjuvant tamoxifen for breast cancer in a randomised trial. The Scottish cancer trials breast group. BMJ 1995;311:977–80. 10.1136/bmj.311.7011.977 7580638 PMC2550987

[24] Rutqvist LE . Long-term toxicity of tamoxifen. Recent Results Cancer Res 1993;127:257–66. 10.1007/978-3-642-84745-5_34 8502824

[25] Bradbury BD , Lash TL , Kaye JA , et al . Tamoxifen-treated breast carcinoma patients and the risk of acute myocardial infarction and newly-diagnosed angina. Cancer 2005;103:1114–21. 10.1002/cncr.20900 15712362

[26] Yang T-L , Wu T-C , Huang C-C , et al . Association of tamoxifen use and reduced cardiovascular events among Asian females with breast cancer. Circ J 2014;78:135–40. 10.1253/circj.CJ-13-0266 24107360

[27] Hernandez RK , Sørensen HT , Jacobsen J , et al . Tamoxifen treatment in Danish breast cancer patients and 5-year risk of arterial atherosclerotic events: a null association. Cancer Epidemiol Biomarkers Prev 2008;17:2509–11. 10.1158/1055-9965.EPI-08-0570 18768523

[28] Ligibel JA , James O'Malley A , Fisher M , et al . Risk of myocardial infarction, stroke, and fracture in a cohort of community-based breast cancer patients. Breast Cancer Res Treat 2012;131:589–97. 10.1007/s10549-011-1754-1 21881937

[29] Geiger AM , Chen W , Bernstein L . Myocardial infarction risk and tamoxifen therapy for breast cancer. Br J Cancer 2005;92:1614–20. 10.1038/sj.bjc.6602562 15841078 PMC2362055

[30] Goss PE , Ingle JN , Martino S , et al . Randomized trial of letrozole following tamoxifen as extended adjuvant therapy in receptor-positive breast cancer: updated findings from NCIC CTG MA.17. J Natl Cancer Inst 2005;97:1262–71. 10.1093/jnci/dji250 16145047

[31] Colleoni M , Giobbie-Hurder A , Regan MM , et al . Analyses adjusting for selective crossover show improved overall survival with adjuvant letrozole compared with tamoxifen in the BIG 1-98 study. J Clin Oncol 2011;29:1117–24. 10.1200/JCO.2010.31.6455 21321298 PMC3083867

[32] Abo-Touk NA , Sakr HA , Abd El-Lattef A . Switching to letrozole versus continued tamoxifen therapy in treatment of postmenopausal women with early breast cancer. J Egypt Natl Canc Inst 2010;22:79–85. 21503010

[33] Kaufmann M , Jonat W , Hilfrich J , et al . Improved overall survival in postmenopausal women with early breast cancer after anastrozole initiated after treatment with tamoxifen compared with continued tamoxifen: the ARNO 95 study. J Clin Oncol 2007;25:2664–70. 10.1200/JCO.2006.08.8054 17563395

[34] Haque R , Shi J , Schottinger JE , et al . Cardiovascular disease after aromatase inhibitor use. JAMA Oncol 2016;2:1590. 10.1001/jamaoncol.2016.0429 27100398

[35] Geiger AM , Fischberg GM , Chen W , et al . Stroke risk and tamoxifen therapy for breast cancer. J Natl Cancer Inst 2004;96:1528–36. 10.1093/jnci/djh285 15494603

[36] Boccardo F , Rubagotti A , Guglielmini P , et al . Switching to anastrozole versus continued tamoxifen treatment of early breast cancer. updated results of the Italian tamoxifen anastrozole (ITA) trial. Ann Oncol 2006;17 Suppl 7:vii10-4. 10.1093/annonc/mdl941 16760270

[37] Bliss JM , Kilburn LS , Coleman RE , et al . Disease-related outcomes with long-term follow-up: an updated analysis of the intergroup Exemestane study. J Clin Oncol 2012;30:709–17. 10.1200/JCO.2010.33.7899 22042946

[38] Schneeweiss S , Patrick AR , Stürmer T , et al . Increasing levels of restriction in pharmacoepidemiologic database studies of elderly and comparison with randomized trial results. Med Care 2007;45:S131–42. 10.1097/MLR.0b013e318070c08e 17909372 PMC2905666

[39] Landon BE , Zaslavsky AM , Saunders RC , et al . Analysis of Medicare advantage HMOs compared with traditional Medicare shows lower use of many services during 2003–09. Health Aff 2012;31:2609–17. 10.1377/hlthaff.2012.0179 PMC3587962 23213144

[40] Landon BE , Zaslavsky AM , Saunders R , et al . A comparison of relative resource use and quality in Medicare advantage health plans versus traditional Medicare. Am J Manag Care 2015;21:559–66. 26295355 PMC6365159

[41] Byhoff E , Harris JA , Ayanian JZ . Characteristics of decedents in Medicare advantage and traditional Medicare. JAMA Intern Med 2016;176:1020–3. 10.1001/jamainternmed.2016.2266 27273237

