# Supplementary material for: Endocrine therapy use and cardiovascular risk in postmenopausal breast cancer survivors
Source: Heart. 2020 Nov 11;107(16):1327–35. doi: 10.1136/heartjnl-2020-317510 (PMC8327412; doi:10.1136/heartjnl-2020-317510)
Supplement: Supplementary data [file heartjnl-2020-317510supp001.pdf]

SUPPLEMENTAL MATERIAL

Section 1 – Supplemental methods

- Appendix 1 - Data source information – p2
- Appendix 2 - Concordance of study populations between UK and US studies – p3
- Appendix 3 - Endocrine therapy billing codes – p4
- Appendix 4 - Ever exposure visualization – p5
- Appendix 5 - CVD outcome billing codes – p6
- Appendix 6 - Covariates adjusted for in UK and US studies – p7
- Appendix 7 - Defining covariates in UK study – p8
- Appendix 8 - Covariate drugs billing codes – p11
- Appendix 9 - Methods to allow direct comparison between UK and US – p12
- Appendix 10 - Methods for quantitative bias analysis to assess potential unmeasured confounding - p13

Section 2 – Supplemental results

- Appendix 11 - Crude rates, unadjusted HRs, and adjusted HRs for the association between ever exposure to endocrine therapies and all CVD outcomes in the UK – p14
- Appendix 12 - Crude rates, unadjusted HRs, and adjusted HRs for the association between ever exposure to endocrine therapies and all CVD outcomes in the US – p15
- Appendix 13 - Adjusted HRs for association between ever exposure to endocrine therapies and risk of CVD, stratified by age, time since index, and prior CVD in the UK – p16
- Appendix 14 - Adjusted HRs for association between ever exposure to endocrine therapies and risk of CVD, stratified by age, time since index, and prior CVD in the US – p17

Section 3 – Supplemental sensitivity analysis

- Appendix 15 - Sensitivity analysis – Adjusted HRs for the association between ever AI compared to ever tamoxifen use and all CVD outcomes in modified analyses in both the UK and US – p18
- Appendix 16 – Sensitivity analysis - Quantitative bias analysis for potential unmeasured confounding results – p19
- Appendix 17 - Adjusted HRs for the association between ever exposure to endocrine therapies and all CVD outcomes in the US, additionally adjusted for adjuvant radiotherapy – p20
- Appendix 18 – Sensitivity analysis - Crude and individually adjusted association between ever exposure to endocrine therapies and all CVD outcomes in the UK – p21
- Appendix 19 – Sensitivity analysis - Crude and individually adjusted association between ever exposure to endocrine therapies and all CVD outcomes in the US – p23

References – p25

Appendix 1 – Data source information

CPRD and HES

The CPRD contains anonymised primary care data from general practitioners (GPs) who agreed at the practice level to participate.[1] CPRD covers 9% of the UK population and is broadly representative of the wider population.[2] The database includes diagnoses, prescriptions and tests from primary care, referrals to specialists, as well as diagnoses and outcomes from secondary care, which are fed back to GPs. Lifestyle and anthropometric measurements are also recorded. Around 60% of patients in the CPRD have linked data available in HES,[3] which includes detailed information on hospitalisations.

SEER-Medicare

The SEER-Medicare database is a linkage of cancer registry and Medicare enrolment and claims data. The SEER program of the US National Cancer Institute is a surveillance system that collates information on demographics, clinical and tumour characteristics, initial surgical and radiation treatment, vital status, and cause of death for all individuals who are diagnosed with cancer and reside within one of the 12 SEER states. The program covers approximately 34.6% of the US population and is representative of the wider US population in terms of poverty and education, but includes more urban areas and a greater proportion of foreign-born residents.[4, 5] The Medicare systems provides governmental funded health insurance for approximately 97% of US citizens aged 65 and over.[6] Medicare health insurance is also available to individuals under 65 if they have been diagnosed with end-stage renal disease or medical disability. As of 2016, 57 million people were covered by Medicare, of whom 48 million were 65 or over (84%). Everyone covered by Medicare is entitled to Part A coverage, which includes hospital inpatient care. Around 96% of people then pay for Part B coverage, which covers physician and outpatient services.[7] 52% of Medicare beneficiaries also have Part D enrolment, which began in 2007 and was available until 2014, and offers outpatient coverage for medications. The SEER-Medicare database is a linkage of the two above databases using a deterministic algorithm based on name, Social security number, sex, and date of birth, and includes cancer cases through 2013 and Medicare claims through 2014.

Matthews AA, *et al. Heart* 2021; 107:1327–1335. doi: 10.1136/heartjnl-2020-317510

Appendix 2 - Concordance of study populations between UK and US studies

	UK study	US study	Concordant	Reason
**Inclusion criteria**				
Gender	Female	Female	✓	
Age	≥54	≥66	X	Only data from those over the age of 65 available in SERR-Medicare
Cancer diagnosis	Breast cancer with one year of follow up prior to diagnosis	ER/PR+ breast cancer with one year of follow up prior to diagnosis	X	ER/PR status not available in CPRD or HES
Date	Breast cancer diagnosis between 1/1/2002 and 31/3/2015	Breast cancer diagnosis between 1/1/2008 and 31/12/2013	X	Medicare Part D follow up started in 2008 and was needed to identify prescriptions
Endocrine therapy prescription	Only women with a tamoxifen or AI prescription after their breast cancer diagnosis	All women with ER/PR+ breast cancer, regardless of if they were prescribed tamoxifen, AIs, or neither. Prescriptions had to be after breast cancer diagnosis	X	As ER/PR status was not available in CPRD or HES, it was impossible to identify an unexposed population with a similar breast cancer diagnosis
**Exclusion criteria**				
Death	At any point prior to index date	At any point prior to index date	✓	
Discontinued follow up in database	At any point prior to index date	At any point prior to index date	✓	
Diagnosed with another cancer	At any point prior to index date	At any point prior to index date	✓	
Diagnosed with CVD event of interest	At any point prior to index date	Within 3 years prior to index date	X	Women are only eligible for Medicare from the age of 66 in the US, so including a longer look back period would mean a greater likelihood of identifying prior events in older women
**Start of follow up**	Latest of 1 year after breast cancer diagnosis or first prescription	1 year after breast cancer diagnosis	X	Under the inclusion criteria in the UK study, women needed a prescription enter study, so couldn't enter prior to this
**Covariates**	Age at index, IMD score, smoking, BMI, alcohol status, statins, ACE inhibitors, CCBs, ARBs, antiplatelets, diabetes, RA, systolic BP, diastolic BP, VTE, non-venous CVD (all CVD outcomes except those within the VTE outcome), year of breast cancer, time since index, current year	Age at index, race, region, statins, ACE inhibitors, CCBs, ARBs, anti-hypertensive drugs, taxanes, anthracyclines, trastuzumab, other systemic therapies, diabetes, RA, hypertension, VTE, non-venous CVDs, year of breast cancer, breast cancer stage, breast cancer grade, time since index, current year	X	All treatments and comorbidities were defined at any point prior to index in the UK, but within 3 years prior to index in the US.<br><br>No lifestyle measures were available in US data.<br><br>No cancer treatment data, other than endocrine therapy, were available in UK data.<br><br>Blood pressure readings were not available in the US, but hypertension and use of anti-hypertensive drugs were adjusted for instead

Appendix 3 - Endocrine therapy billing codes

	**HCPCS**	**NDC**
**Tamoxifen**	S0187	00038060025, 00038060060, 00054483121, 00054483126, 00054483413, 00054483422, 00054883125, 00054883425, 00093078210, 00093078256, 00093078405, 00093078406, 00093078410, 00172565658, 00172565670, 00172565680, 00172565746, 00172565755, 00172565760, 00172565770, 00310060018, 00310060075, 00310060412, 00310060430, 00310060490, 00310073060, 00378014405, 00378014491, 00378027401, 00378027493, 00440845092, 00440845130, 00440845160, 00440845192, 00555044603, 00555044605, 00555044609, 00555044663, 00555090401, 00591223218, 00591223319, 00591223330, 00591247319, 00591247330, 38779034101, 38779034103, 38779034108, 49452775301, 51552083802, 51927297600, 53002103203, 53002103230, 54569038201, 54569038202, 54569376501, 54569571600, 54569853100, 54569860200, 54868300401, 54868300402, 54868300403, 54868300404, 54868428700, 54868428703, 54868428704, 57866661501, 58016065760, 60346004832, 62991115101, 62991115103, 63304060060, 63304060130, 63304060190, 63739026910, 63739026915, 00093078201, 00093078205, 00093078486, 00172565649, 00172565780, 00310060025, 00310060060, 00310073130, 00440845030, 00440845060, 00555090405, 00555090414, 00591223260, 00591247218, 00591247260, 13632012301, 38779034104, 38779034105, 42254034390, 52372075601, 52372075602, 54569038200, 54569376500, 54868300405, 54868428701, 54868428702, 55175550006, 55289058530, 57866661801, 62991115104, 63304060028
**Aromatase inhibitors**	S0170<br><br>S0156	Anastrozole:<br><br>00054016413, 00093753656, 00310020130, 00310020137, 00310020150, 00378603405, 00378603477, 00781535631, 00904619546, 00904622961, 16571042103, 16729003510, 16729003515, 16729003516, 21695099030, 35356027030, 38779227406, 38779255503, 38779255504, 38779255506, 42043018003, 42291010530, 51079032301, 51079032306, 51927443500, 51991062010, 51991062033, 54569573100, 54569619800, 54868500000, 54868613000, 54868613001, 55111064730, 55175550503, 60258086603, 62756025013, 62756025083, 63275993001, 63275993002, 63323012930, 66435041530, 67877017110, 67877017130, 68084044811, 68084044821, 68258903501, 68382020906, 42254016130, 60429028630, 60429028690, 60505298503, 63275993003, 63275993004, 63275993005, 68382020910<br><br>Exemestane:<br><br>00054008013, 49999098630, 54569573200, 54868526100, 59762285801, 00009766304<br><br>Letrozole:<br><br>00054026913, 00078024915, 00093762056, 00378207105, 00378207193, 00603418016, 16729003410, 35356040930, 51991075910, 51991075933, 54569571400, 54868415100, 54868625200, 55111064630, 62756051183, 63323077230<br><br>42254024330, 60505325503, 60505325508

Matthews AA, *et al. Heart* 2021; 107:1327–1335. doi: 10.1136/heartjnl-2020-317510

Appendix 4 - Ever exposure visualisation

UK

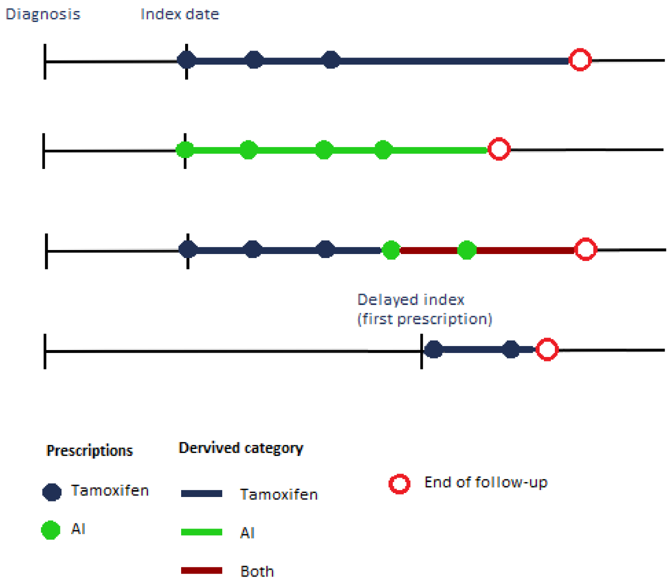

Further explanation: All women were exposed to either tamoxifen or AIs at index date. Their index date was the latest of either 1 year after breast cancer diagnosis, or first endocrine therapy prescription (the distinction between first three scenarios and the fourth scenario). Once a woman started follow up, they were considered ever exposed to either a tamoxifen or AI. They continued to be exposed to this until they either switched therapy (in which case their exposure was updated to indicate they had ever been exposed to both endocrine therapies) or the end of follow up.

US

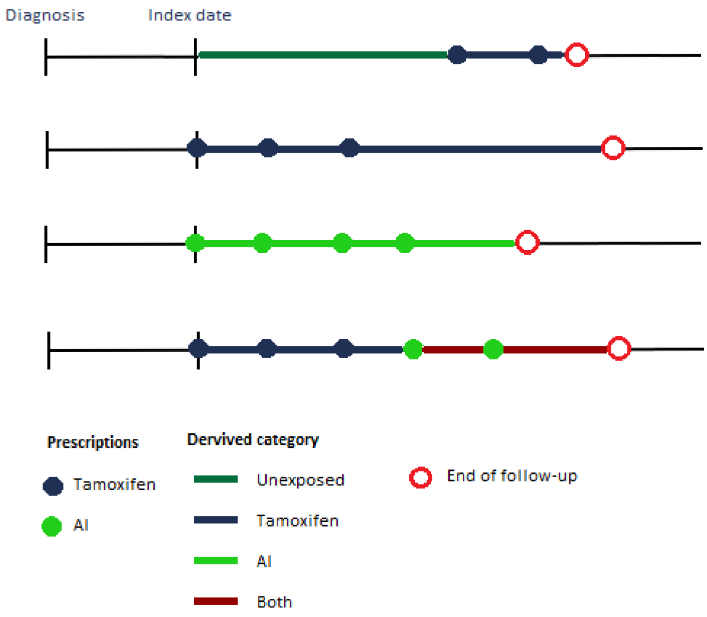

Further explanation: Ever exposure was set up in the same way in the US study as in the UK study above, except, if women were not exposed to any endocrine therapy one year after breast cancer diagnosis, they were categorised as unexposed. They continued to be unexposed until either the end of follow up or the point at which they were prescribed an endocrine therapy.

Matthews AA, *et al. Heart* 2021; 107:1327–1335. doi: 10.1136/heartjnl-2020-317510

Appendix 5 - CVD outcome billing codes

Composite outcomes	Individual outcomes	ICD 9 diagnosis and procedure codes	CPT/HCPCS codes
Coronary Artery Disease	Angina	411.1, 413.1, 413.9	
	Myocardial infarction	410.11, 410.01, 410.31, 410.21, 410.41, 410.81, 410.51, 410.61, 410.91, 410.71	
	Revascularization procedures	36.0x, 36.1x, 36.2, 36.3	33140, 33510, 33511, 33512, 33513, 33514, 33516, 33517, 33518, 33519, 33521, 33522, 33523, 33533, 33534, 33535, 33536, 92920, 92924, 92928, 92933, 92937, 92941, 92943, 92980, 92981, 92982, 92984, 92995, 92996, 92997, 92998
	Sudden cardiac arrest	427.5	
	Peripheral vascular disease	443.89, 443.9	
	Stroke (haemorrhagic and ischaemic)	430.x, 431.x, 432.1, 432.0, 432.9, 433.91, 433.21, 433.01, 433.11, 433.31, 433.81, 434.01, 434.x, 435.0,<br>435.1, 435.3, 435.8, 435.9, 436.x	
	Arrhythmia	427.31, 427.32, 427.41, 427.42, 427.61, 427.0, 427.69, 427.60, 427.81, 427.89, 427.9	
	Heart failure	HF - 428.1, 428.0, 428.20, 428.21, 428.22, 428.23, 428.30, 428.31, 428.32, 428.33, 428.40, 428.41, 428.42, 428.43, 428.9<br><br>Cardiomyopathy - 414.8, 425.4, 425.11, 425.18, 425.0, 425.3, 425.5, 425.9, 425.2, 425.8	
	Pericarditis	420.91, 420.90, 420.99, 423.1, 423.2, 423.0, 423.9, 423.3, 423.8 420.0	
	Valvular heart disease	394.0, 394.1, 394.2, 394.9, 395.0, 395.1, 395.2, 395.9, 397.0, 396.0, 396.1, 396.2, 396.3, 397.9, 396.8, 396.9, 424.0, 424.1, 424.2, 424.3	
VTE	Deep venous thromboembolism	415.1x	
	Pulmonary Embolism	415.0, 415.12, 415.13, 415.19	

Matthews AA, *et al. Heart* 2021; 107:1327–1335. doi: 10.1136/heartjnl-2020-317510

Appendix 6 - Covariates adjusted for in UK and US studies

Covariate	UK study		US study	
	Adjusted	Definition	Adjusted	Definition
Demographic				
Age at index	✓	54-59, 60-69, 70+	✓	66-74, 75-84, 85+
IMD score	✓	level 1-5 based on GP level IMD data	X	
Race	X		✓	White, Black Asian, Hispanic, Native American, other
Region	X		✓	North East, South, North Central, West
Lifestyle measures				
Smoking status	✓	At index (non-smoker, current smoker, ex-smoker)	X	
BMI	✓	At index (underweight/healthy weight, overweight, obese)	X	
Alcohol status	✓	At index (non-drinker, current drinker, ex-drinker)	X	
Treatments				
Use of statins	✓	Ever use prior to index	✓	Use within 3-years prior to index
Use of ACE inhibitors	✓	Ever use prior to index	✓	Use within 3-years prior to index
Use of CCBs	✓	Ever use prior to index	✓	Use within 3-years prior to index
Use of ARBs	✓	Ever use prior to index	✓	Use within 3-years prior to index
Use of antiplatelets	✓	Ever use prior to index	X	
Use of anti-hypertensive drugs	X		✓	Use within 3-years prior to index
Use of taxanes	X		✓	Use within 3-years prior to index
Use of anthracyclines	X		✓	Use within 3-years prior to index
Use of trastuzumab	X		✓	Use within 3-years prior to index
Use of other systemic cancer therapies	X		✓	Use within 3-years prior to index
Comorbidity diagnoses				
Diabetes	✓	Ever diagnosed prior to index	✓	Diagnosed within 3-years prior to index
Rheumatoid arthritis	✓	Ever diagnosed prior to index	✓	Diagnosed within 3-years prior to index
Systolic blood pressure	✓	At index (low/normal, pre-high, high)	X	
Diastolic blood pressure	✓	At index (low/normal, pre-high, high)	X	
Hypertension	X	N/A	✓	Diagnosed within 3-years prior to index
History of non-venous CVD	✓	Ever diagnosed prior to index	✓	Diagnosed within 3-years prior to index
History of any VTE outcome	✓	Ever diagnosed prior to index	✓	Diagnosed within 3-years prior to index
Information relating to breast cancer				
Year of breast cancer diagnosis	✓	2002-2015	✓	2007-2013
Breast cancer stage	X		✓	1-3
Breast cancer grade	X		✓	1-3
Other information				
Time since index date	✓	0-1yr, 1-3yrs, 3-5yrs, 5+yrs	✓	0-1yr, 1-3yrs, 3-5yrs, 5+yrs
Current year	✓	2002-2017	✓	2007-2014

Matthews AA, *et al. Heart* 2021; 107:1327–1335. doi: 10.1136/heartjnl-2020-317510

Appendix 7 – Defining covariates in UK study

All code lists are available at https://doi.org/10.17037/DATA.177.

Diagnoses

Diabetes and rheumatoid arthritis
Read code lists were used to search CPRD clinical and referral files to identify any women in the study population with a diabetes or rheumatoid arthritis diagnosis prior to index date.

Chronic kidney disease
Records of an eGFR reading by GPs were identified in the CPRD additional files. CKD was then established by calculating eGFR using the Chronic Kidney Disease Epidemiology Collaboration equation.[8] Serum creatinine measurements were not routinely isotope-dilution mass spectrometry-standardised until 2013. It was therefore assumed that all creatinine results were unstandardized and multiplied results with a correction factor of 0.95 before calculating eGFR without regard to ethnicity.[9] To avoid selection bias, an absent CKD category was included for those with no recorded serum creatinine result. The recording closest to, but before index date was used.

Systolic and diastolic blood pressure
CPRD Additional files were searched for instances of when blood pressure was recorded by the GP. The following algorithm to identified blood pressure records:

- Drop record if systolic or diastolic <30 or >250
- Drop if the record indicates it is a target blood pressure
- Drop a duplicate if they indicate the same reading on the same day
- If there are more than once sensible reading in the same day, calculate the mean

The following algorithm then assigned a systolic and diastolic blood pressure recording to all women with relevant records:

- Take the nearest status in the period -1y to +1month from index if available (best option)
- if not, then take nearest in the period +1month to +1y after index if available (second best option)
- if not, then take any nearest before -1y from index if available (third best option) if not, then take nearest after +1y from index (least best option)

Blood pressures were then categorised into the following categories.

- Systolic – low and ideal (<120), pre-high (>=120 & <140), high (>=140)
- Diastolic – low and ideal (<80), pre-high (>=80 & <90), high (>=90)

Prescriptions

Statins, ACE inhibitors, calcium channel blockers, angiotensin II receptor blockers, and anti-platelets
Product code lists were created to search the CPRD therapy files to identify women in the study population with a prescription of any of these drugs prior to index date.

Lifestyle measures

Smoking status
A Read code list was created to search for all records of a smoking status in the CPRD clinical file. Further available data from the additional file were also extracted, including the patients smoking “status” (yes, no or

Matthews AA, *et al. Heart* 2021; 107:1327–1335. doi: 10.1136/heartjnl-2020-317510

ex) and the "number of cigarettes per day" smoked. The following algorithm was then used to assign a smoking status to all women with relevant records.

- Take the nearest status in the period -1y to +1month from index if available (best option)
- if not, then take nearest in the period +1month to +1y after index if available (second best option)
- if not, then take any nearest before -1y from index if available (third best option)
- if not, then take nearest after +1y from index (least best option)

Smoking status was then categorised into non-smoker, current smoker, and ex-smoker.

Alcohol status
A Read code list was created to identify all records of alcohol usage in the CPRD clinical file. Further available data from the additional file were also extracted, including the patient's alcohol drinking "status" (yes, no or ex) and the "units per week" consumed. The above algorithm used to define smoking status was also used to assign an alcohol status to all women with relevant records.

Alcohol status was then categorised into non-drinker, current drinker, ex-drinker

BMI
CPRD additional files were searched for records of women's height and weight. BMI is calculated using patients' weight in kilograms / (height in meters)^2. The following algorithm, which was described in the paper by Bhaskaran et al. on BMI in the CPRD,[10] was used to identify for BMI records:

- drop if 3+ measurements on the same day
- if 2 measurements on same day: drop if >5cm (ht)/1kg (wt) difference, otherwise take the mean
- initial pass, drop weights less than 2kg, heights less than 2 feet
- drop records after end of follow-up but keeps those before start of follow-up
- later, drops weights < 20kg, heights less than 4 or more than 7 feet
- fill in missing heights using last observation carried forward or if no previous, first future height measurement
- calculate a version of BMI directly from height and weight
- drop BMIs <5 or >200 (but if CPRD and calculated version differ, and one is in the range 10-100, uses the sensible one)
- in general, prioritises calculated BMI, and only uses CPRD version if cannot be calculated (as no height measurement available at all)

The above algorithm used to assign smoking status was then used to assign BMI status to all women with relevant records.

BMI was categorised according to adult BMI cut-offs defined by the WHO, as underweight or healthy weight (BMI <24.9), overweight (BMI 25+), or obese (BMI ≥30).

Matthews AA, *et al. Heart* 2021; 107:1327–1335. doi: 10.1136/heartjnl-2020-317510

Demographic measures

Age

As CPRD only supplies year of birth, the date of birth was set to 1st of July for all women. Age at index was then calculated and categorised into 54-60, 61-70, 70+.

Index of multiple deprivation (IMD)

CPRD supplies patient level IMD scores at the practice level for all patients. IMD combines a number of indicators, chosen to cover a range of economic, social and housing issues, into a single deprivation score.[11] This score was consolidated into quintiles, with a low quintile representing the least deprived, and a high quintile representing the most deprived.

Appendix 8 - Covariate drugs billing codes

**Chemotherapy billing codes**	
**Drug Class**	**HCPCS/CPT *(variable)***
Taxanes	C9127, C9431, J9170, J9171, J9264, J9265, Q0125
Anthracyclines	C9415, J9000, J9001, J9178, J9180
Trastuzumab	J9355
Others	96408, 96409, 96410, 96411, 96412, 96413, 96414, 96415, 96416, 96417, 96545, 96549, C1167, C8953, C8954, C8955, C9214, C9240, C9257, C9280, C9414, C9418, C9420, C9421, C9425, C9438, C9440, G0359, G0360, G9021, G9022, G9023, G9024, G9025, G9026, G9027, G9028, G9029, G9030, G9031, G9032, J8520, J8521, J8530, J8560, J8999, J9035, J9045, J9060, J9062, J9070, J9080, J9090, J9091, J9092, J9093, J9094, J9095, J9096, J9097, J9179, J9181, J9182, J9190, J9201, J9207, J9250, J9260, J9293, J9360, J9390, J9999, Q0083, Q0084, Q0085
**Concomitant medication billing codes**	
**Drug class**	**ATC codes**
Hypertensives	C02
Statins	C10AA (C10AA01-C10AA08)
Ace Inhibitors	C09AA (C09AA01-C09AA16)
Calcium Channel Blockers	C08CA (C08CA01-C08CA16, C08CA55)
Angiotensin 2 receptor blocker	C09CA (C09CA01-C09CA09)

Matthews AA, *et al. Heart* 2021; 107:1327–1335. doi: 10.1136/heartjnl-2020-317510

Appendix 9 - Methods to allow direct comparison between UK and US

In general, the UK study population was restricted to those aged ≥65 years to match the US data, while the US study population was restricted to those prescribed endocrine therapy as in the UK study; in addition, analyses were adjusted only for covariates available in both settings. Full details on the study populations, exposures, outcomes, covariates, and statistical analyses follow.

Study populations
The study populations were modified for both studies to include all women with CPRD and HES/Medicare Parts A, B and D coverage the 12-months before an incident breast cancer diagnosis. All women had to be over the age of 65, with an incident ER/PR+ and stage 1-3 breast cancer (in the US study, any breast cancer in the UK study) diagnosed between 1st January 2002 and to 31st March 2016 in the UK, and 1st January 2008 and 31st December 2013 in the US, and be newly prescribed an AI or tamoxifen after their diagnosis. In addition, women were not allowed to have an endocrine therapy prescription prior to their breast cancer diagnosis. Follow-up began either one year after the date of breast cancer or at the date of first AI or tamoxifen prescription, whichever occurred latest (hereafter the 'index date'). Women were excluded if prior to their index date they: died, discontinued from Medicare Parts A, B, or D/transferred out of the CPRD, were diagnosed with any cancer relating to sites other than the breast, or were diagnosed with the CVD event of interest (within a 3-year period prior to index).

Exposures
In both UK and US study populations, incident tamoxifen and AI exposures were identified using an appropriate prescription code (based on the code lists outlined in the main methods). The primary exposure was AI use relative to tamoxifen use. We considered ever exposure to endocrine therapy (ever use of tamoxifen, ever use of AI, ever use of both drugs). If a woman moved between tamoxifen and AI prescriptions, records were time-updated to indicate they had ever been exposed to both drugs from this point forward.

Outcomes
All CVD outcomes explored in both the UK and US studies continued to be the main CVD outcomes of interest for this comparison analysis. Events were identified using Read/ICD-10 codes in CPRD/HES respectively, and ICD-9 codes in SEER-Medicare.

Covariates
Covariates that were adjusted for in both analyses were: year of breast cancer diagnosis (2007-2013); age at index date (65-75, 75-85, 85+); time since index date (0-1yr, 1-3yrs, 3-5yrs, 5+yrs); and current calendar year; use of statins; use of ACE inhibitors; use of calcium channel blockers; use of angiotensin receptor blockers; rheumatoid arthritis; chronic kidney disease; diabetes; VTE; and non-venous CVD. Identification of diagnoses and prescriptions were restricted to a 3-year look back window prior to index date.

Statistical Analysis
For both studies, observation time began at index date and ended at earliest of the following: a CVD event of interest, diagnosis of another cancer, death, transfer out of the CPRD network/end of enrolment in Medicare Parts A, B, or D, or end of follow-up (31st March 2016 in the UK, 31st December 2014 in the US). Prior to exploring the relationship between endocrine therapies and CVD, baseline characteristic distributions of patients who were initially prescribed tamoxifen or AIs were described. The primary exposure was then included in unadjusted and adjusted (accounting for all covariates) Cox regression models with an underlying age timescale, to obtain hazard ratios.

Matthews AA, *et al. Heart* 2021; 107:1327–1335. doi: 10.1136/heartjnl-2020-317510

Appendix 10 – Methods for quantitative bias analysis to assess potential unmeasured confounding

Results suggested a decreased risk of several CVD outcomes in tamoxifen users compared with those unexposed to any endocrine therapy. However, we believe that residual confounding may be present due to reasons for reasons for non-initiation of endocrine therapy, such as frailty, poor CVD preventative care, and high BMI. We therefore explored the effect that such residual confounding could have on our effect estimates.[12] To quantify the potential residual confounding, we require the association between the confounder and outcome, and the prevalence of the confounder in each level of the exposure. As an example, we aimed to quantify the potential effect of residual confounding by frailty on the association between ever tamoxifen use compared with those unexposed to endocrine therapy, and the risk of MI. Basic bias analysis for unmeasured confounding were carried out using the spreadsheets available at: https://sites.google.com/site/biasanalysis/.

Given assumptions about the distribution of frailty in the population and the effect of frailty on the outcome MI in the absence of the exposure, the adjusted risk ratio can be expressed as:

$$RR_{adj} = RR_{obs}\frac{RR_{CD}p_0 + (1 - p_0)}{RR_{CD}p_1 + (1 - p_1)}$$

where $\mathrm{RR}_{\mathrm{adj}}$ is the risk ratio associating endocrine therapy use with MI adjusted for frailty, $\mathrm{RR}_{\mathrm{obs}}$ is the observed risk ratio associating endocrine therapy use with the MI without adjustment for the frailty, RR_{CD} is the risk ratio associating the frailty with MI (assuming no effect measure modification by the exposure), and p_1 and p_0 are the proportions of subjects with frailty in the exposed and unexposed groups respectively.

Matthews AA, *et al. Heart* 2021; 107:1327–1335. doi: 10.1136/heartjnl-2020-317510

Appendix 11 - Crude rates, unadjusted HRs, and adjusted HRs for the association between ever exposure to endocrine therapies and all CVD outcomes in the UK

Outcome	Ever exposure	Number of events	Follow up (per 1000 pyears)	Rate (95% CI, per 1000 pyears)	Age-standardised rate (95% CI, per 1000 pyears)	Unadjusted HR (95% CI)	Adjusted HR (95% CI) *
**Coronary Artery Disease**	Tamoxifen	93	13.54	6.87 (5.60, 8.41)	7.51 (5.97, 9.05)	1	1
	AI	131	12.87	10.18 (8.58, 12.08)	10.17 (8.42, 11.91)	1.40 (1.06, 1.86)	1.29 (0.94, 1.76)
	Both	67	10.6	6.32 (4.98, 8.03)	7.22 (5.46, 8.97)	0.95 (0.69, 1.31)	0.95 (0.68, 1.32)
Angina	Tamoxifen	56	13.73	4.08 (3.14, 5.30)	4.35 (3.20, 5.50)	1	1
	AI	80	13.15	6.09 (4.89, 7.58)	6.03 (4.70, 7.35)	1.43 (1.00, 2.05)	1.31 (0.88, 1.97)
	Both	31	10.73	2.89 (2.03, 4.11)	3.18 (2.05, 4.31)	0.70 (0.44, 1.10)	0.69 (0.43, 1.10)
MI	Tamoxifen	32	14.6	2.19 (1.55, 3.10)	2.55 (1.66, 3.44)	1	1
	AI	61	14.11	4.32 (3.36, 5.56)	4.34 (3.24, 5.43)	1.73 (1.12, 2.68)	1.56 (0.96, 2.52)
	Both	39	11.24	3.47 (2.54, 4.75)	4.39 (2.97, 5.80)	1.62 (1.01, 2.60)	1.62 (0.99, 2.63)
Revascularisation	Tamoxifen	15	14.72	1.02 (0.61, 1.69)	0.98 (0.48, 1.47)	1	1
	AI	20	14.3	1.40 (0.90, 2.17)	1.36 (0.76, 1.96)	1.36 (0.69, 2.68)	1.84 (0.85, 4.02)
	Both	12	11.33	1.06 (0.60, 1.86)	1.02 (0.44, 1.60)	0.99 (0.46, 2.11)	1.01 (0.46, 2.22)
SCA	Tamoxifen	13	14.88	0.87 (0.51, 1.50)	0.94 (0.43, 1.46)	1	1
	AI	21	14.53	1.45 (0.94, 2.22)	1.44 (0.82, 2.06)	1.87 (0.83, 4.22)	1.65 (0.65, 4.19)
	Both	5	11.43	0.44 (0.18, 1.05)	0.49 (0.06, 0.93)	0.73 (0.24, 2.19)	0.68 (0.22, 2.09)
**PVD**	Tamoxifen	35	14.64	2.39 (1.72, 3.33)	2.66 (1.76, 3.55)	1	1
	AI	41	14.08	2.91 (2.14, 3.96)	2.94 (2.04, 3.85)	1.25 (0.77, 2.01)	1.31 (0.76, 2.25)
	Both	22	11.23	1.96 (1.29, 2.97)	2.30 (1.32, 3.28)	0.81 (0.45, 1.43)	0.86 (0.48, 1.57)
**Stroke**	Tamoxifen	91	14.45	6.30 (5.13, 7.73)	7.10 (5.63, 8.58)	1	1
	AI	118	13.71	8.61 (7.19, 10.31)	8.76 (7.17, 10.35)	1.22 (0.91, 1.63)	1.11 (0.81, 1.52)
	Both	88	10.98	8.02 (6.51, 9.88)	9.77 (7.67, 11.87)	1.33 (0.98, 1.80)	1.25 (0.91, 1.71)
**Arrhythmia**	Tamoxifen	219	12.7	17.25 (15.11, 19.69)	19.27 (16.69, 21.85)	1	1
	AI	287	11.52	24.90 (22.18, 27.96)	25.53 (22.57, 28.49)	1.37 (1.14, 1.64)	1.37 (1.11, 1.68)
	Both	174	9.51	18.30 (15.77, 21.23)	21.76 (18.44, 25.07)	1.07 (0.87, 1.32)	1.10 (0.89, 1.36)
**HF**	Tamoxifen	90	14.33	6.28 (5.11, 7.72)	7.57 (5.99, 9.15)	1	1
	AI	178	13.59	13.10 (11.31, 15.17)	13.59 (11.58, 15.61)	1.87 (1.43, 2.45)	1.68 (1.24, 2.26)
	Both	76	11.04	6.89 (5.50, 8.62)	9.32 (7.17, 11.48)	1.12 (0.81, 1.55)	1.12 (0.80, 1.56)
**Pericarditis**	Tamoxifen	3	14.85	0.20 (0.07, 0.63)	0.20 (-0.03, 0.43)	1	1
	AI	14	14.53	0.96 (0.57, 1.63)	0.97 (0.46, 1.48)	3.96 (1.12, 14.03)	3.25 (0.86, 12.23)
	Both	10	11.4	0.88 (0.47, 1.63)	0.86 (0.32, 1.41)	4.29 (1.18, 15.61)	3.57 (0.95, 13.50)
**VHD**	Tamoxifen	66	14.54	4.54 (3.57, 5.78)	5.22 (3.95, 6.49)	1	1
	AI	114	13.93	8.18 (6.81, 9.83)	8.29 (6.76, 9.82)	1.58 (1.15, 2.17)	1.30 (0.92, 1.85)
	Both	52	11.14	4.67 (3.56, 6.13)	5.86 (4.23, 7.49)	1.08 (0.74, 1.57)	0.98 (0.67, 1.43)
**VTE**	Tamoxifen	122	14.41	8.47 (7.09, 10.11)	9.08 (7.45, 10.71)	1	1
	AI	116	13.4	8.66 (7.22, 10.38)	8.65 (7.07, 10.22)	0.93 (0.71, 1.21)	0.82 (0.61, 1.10)
	Both	85	10.63	8.00 (6.47, 9.89)	8.97 (7.03, 10.92)	0.91 (0.69, 1.22)	0.95 (0.71, 1.28)
DVT	Tamoxifen	83	14.52	5.72 (4.61, 7.09)	6.26 (4.90, 7.63)	1	1
	AI	62	13.78	4.50 (3.51, 5.77)	4.49 (3.37, 5.61)	0.69 (0.48, 0.98)	0.63 (0.42, 0.92)
	Both	59	10.83	5.45 (4.22, 7.03)	6.32 (4.67, 7.97)	0.98 (0.69, 1.38)	1.04 (0.73, 1.49)
PE	Tamoxifen	48	14.75	3.25 (2.45, 4.32)	3.41 (2.44, 4.38)	1	1
	AI	68	14.06	4.84 (3.81, 6.13)	4.84 (3.69, 5.99)	1.39 (0.95, 2.04)	1.21 (0.79, 1.85)
	Both	33	11.19	2.95 (2.10, 4.15)	3.18 (2.08, 4.27)	0.80 (0.51, 1.28)	0.78 (0.48, 1.25)

*Adjusted the following covariates at baseline: for age (54-59, 60-69, 70+); smoking status (non-smoker, current smoker, ex-smoker); BMI (underweight/healthy weight, overweight, obese); alcohol status (non-drinker, current drinker, ex-drinker); IMD score (level 1-5 based on GP level IMD data); use of statins; use of ACE inhibitors; use of calcium channel blockers (CCB); use of angiotensin II receptor blockers (ARB); diabetes; chronic kidney disease; rheumatoid arthritis; systolic blood pressure (low/normal, pre-high, high); diastolic blood pressure (low/normal, pre-high, high); history of VTE; history of non-venous CVD year of breast cancer diagnosis; time since index (0-1yrs, 1-3yrs, 3-5yrs, 5+yrs); and current year.

Matthews AA, *et al. Heart* 2021; 107:1327–1335. doi: 10.1136/heartjnl-2020-317510

Appendix 12 - Crude rates, unadjusted HRs, and adjusted HRs for the association between ever exposure to endocrine therapies and all CVD outcomes in the US

Outcome	Ever exposure	Events, Follow-up* (per 1000 pyears)	Rate (95% CI, per 1000 pyears)	Age-standardised rate (95% CI, per 1000 pyears)	Unadjusted HR (95% CI)	Adjusted HR (95% CI)**	Adjusted HR (95% CI)***
**Coronary Artery Disease**	Unexposed	318, 8.22	38.70 (34.67, 43.19)	35.19 (31.25, 39.12)	1	1	1.34 (1.09, 1.66)
	Tamoxifen	129, 4.79	26.95 (22.68, 32.03)	26.02 (21.51, 30.54)	0.74 (0.60, 0.91)	0.74 (0.60, 0.92)	1
	AI	1059, 30.03	35.26 (33.20, 37.45)	36.82 (34.57, 39.06)	1.01 (0.89, 1.15)	0.96 (0.83, 1.10)	1.29 (1.06, 1.55)
	Both	118, 3.87	30.51 (25.47, 36.54)	32.06 (26.26, 37.85)	0.86 (0.69, 1.07)	0.89 (0.71, 1.10)	1.19 (0.92, 1.54)
Angina	Unexposed	189, 8.40	22.51 (19.52, 25.96)	21.54 (18.42, 24.66)	1	1	1.13 (0.87, 1.46)
	Tamoxifen	87, 4.83	18.00 (14.59, 22.21)	17.69 (13.97, 21.41)	0.85 (0.66, 1.10)	0.88 (0.68, 1.14)	1
	AI	710, 30.61	23.20 (21.55, 24.97)	23.62 (21.86, 25.38)	1.06 (0.90, 1.26)	1.05 (0.89, 1.25)	1.19 (0.95, 1.50)
	Both	76, 3.97	19.13 (15.28, 23.95)	19.50 (15.11, 23.89)	0.89 (0.68, 1.17)	0.95 (0.72, 1.25)	1.07 (0.78, 1.46)
MI	Unexposed	153, 9.39	16.30 (13.91, 19.10)	14.14 (11.86, 16.43)	1	1	2.29 (1.58, 3.33)
	Tamoxifen	38, 5.41	7.02 (5.11, 9.65)	6.75 (4.60, 8.90)	0.45 (0.31, 0.65)	0.44 (0.30, 0.63)	1
	AI	407, 34.29	11.87 (10.77, 13.08)	12.68 (11.43, 13.93)	0.90 (0.74, 1.10)	0.79 (0.64, 0.97)	1.81 (1.28, 2.58)
	Both	42, 4.35	9.66 (7.14, 13.07)	10.44 (7.28, 13.61)	0.71 (0.50, 1.01)	0.67 (0.47, 0.96)	1.53 (0.97, 2.42)
Revascularisation	Unexposed	65, 9.40	6.91 (5.42, 8.81)	7.19 (5.42, 8.96)	1	1	1.60 (0.99, 2.57)
	Tamoxifen	25, 5.37	4.66 (3.15, 6.89)	4.64 (2.82, 6.46)	0.65 (0.41, 1.05)	0.63 (0.39, 1.01)	1
	AI	234, 34.29	6.82 (6.00, 7.76)	6.72 (5.85, 7.58)	0.92 (0.69, 1.23)	0.91 (0.68, 1.23)	1.46 (0.95, 2.24)
	Both	30, 4.33	6.94 (4.85, 9.92)	6.77 (4.34, 9.19)	0.93 (0.60, 1.46)	0.99 (0.63, 1.55)	1.57 (0.91, 2.72)
SCA	Unexposed	80, 9.70	8.25 (6.63, 10.27)	7.04 (5.47, 8.62)	1	1	1.49 (0.96, 2.33)
	Tamoxifen	29, 5.50	5.28 (3.67, 7.59)	5.08 (3.23, 6.92)	0.69 (0.44, 1.07)	0.67 (0.43, 1.04)	1
	AI	222, 35.21	6.31 (5.53, 7.19)	6.79 (5.88, 7.70)	0.91 (0.69, 1.20)	0.78 (0.59, 1.04)	1.17 (0.78, 1.76)
	Both	28, 4.48	6.25 (4.32, 9.05)	6.79 (4.27, 9.32)	0.82 (0.52, 1.31)	0.80 (0.50, 1.28)	1.20 (0.69, 2.08)
**PVD**	Unexposed	331, 7.81	42.40 (38.07, 47.23)	37.56 (33.45, 41.67)	1	1	1.10 (0.91, 1.34)
	Tamoxifen	158, 4.56	34.64 (29.64, 40.48)	33.30 (28.10, 38.49)	0.89 (0.74, 1.08)	0.91 (0.75, 1.10)	1
	AI	1075, 28.85	37.26 (35.10, 39.55)	39.44 (37.05, 41.83)	1.05 (0.92, 1.19)	1.00 (0.87, 1.14)	1.10 (0.92, 1.31)
	Both	129, 3.81	33.85 (28.48, 40.22)	35.89 (29.68, 42.10)	0.91 (0.73, 1.12)	0.93 (0.75, 1.15)	1.02 (0.80, 1.30)
**Stroke**	Unexposed	404, 7.66	52.76 (47.86, 58.16)	45.52 (41.00, 50.03)	1	1	1.22 (1.02, 1.45)
	Tamoxifen	190, 4.65	40.85 (35.43, 47.09)	39.17 (33.60, 44.75)	0.85 (0.71, 1.02)	0.82 (0.69, 0.98)	1
	AI	1126, 28.82	39.07 (36.85, 41.42)	42.09 (39.59, 44.59)	0.93 (0.82, 1.05)	0.87 (0.76, 0.98)	1.05 (0.90, 1.24)
	Both	134, 3.72	36.06 (30.44, 42.71)	39.10 (32.46, 45.74)	0.84 (0.69, 1.03)	0.81 (0.66, 1.00)	0.99 (0.79, 1.24)
**Arrhythmia**	Unexposed	510, 5.97	85.40 (78.30, 93.14)	78.10 (71.20, 84.99)	1	1	1.34 (1.14, 1.58)
	Tamoxifen	222, 3.67	60.49 (53.04, 69.00)	58.64 (50.92, 66.36)	0.73 (0.62, 0.86)	0.74 (0.63, 0.88)	1
	AI	1640, 22.80	71.92 (68.52, 75.48)	75.06 (71.37, 78.74)	0.94 (0.85, 1.05)	0.91 (0.81, 1.01)	1.22 (1.05, 1.41)
	Both	189, 2.87	65.90 (57.14, 76.00)	68.85 (59.01, 78.68)	0.85 (0.71, 1.01)	0.88 (0.74, 1.04)	1.17 (0.96, 1.43)
**HF**	Unexposed	488, 6.77	72.07 (65.95, 78.76)	61.45 (55.91, 66.98)	1	1	1.15 (0.98, 1.36)
	Tamoxifen	233, 4.17	55.90 (49.16, 63.56)	53.66 (46.78, 60.55)	0.85 (0.72, 1.00)	0.87 (0.74, 1.02)	1
	AI	1368, 26.03	52.56 (49.85, 55.42)	57.17 (54.09, 60.26)	0.93 (0.83, 1.04)	0.84 (0.75, 0.94)	0.96 (0.83, 1.12)
	Both	167, 3.44	48.56 (41.72, 56.51)	54.04 (45.81, 62.27)	0.85 (0.71, 1.02)	0.83 (0.69, 1.00)	0.96 (0.78, 1.18)
**Pericarditis**	Unexposed	74, 9.48	7.80 (6.21, 9.80)	7.47 (5.73, 9.21)	1	1	2.69 (1.54, 4.71)
	Tamoxifen	16, 5.44	2.94 (1.80, 4.80)	2.91 (1.48, 4.33)	0.37 (0.21, 0.64)	0.37 (0.21, 0.65)	1
	AI	197, 34.48	5.71 (4.97, 6.57)	5.82 (5.00, 6.64)	0.74 (0.56, 0.98)	0.67 (0.50, 0.90)	1.81 (1.06, 3.08)
	Both	20, 4.43	4.52 (2.91, 7.00)	4.61 (2.59, 6.64)	0.55 (0.33, 0.93)	0.57 (0.34, 0.96)	1.53 (0.77, 3.05)
**VHD**	Unexposed	447, 6.08	73.51 (67.00, 80.65)	68.04 (61.64, 74.45)	1	1	1.24 (1.05, 1.47)
	Tamoxifen	205, 3.69	55.62 (48.50, 63.78)	53.75 (46.39, 61.12)	0.78 (0.66, 0.93)	0.81 (0.68, 0.96)	1
	AI	1513, 22.37	67.63 (64.31, 71.13)	70.23 (66.65, 73.81)	1.01 (0.90, 1.13)	0.98 (0.87, 1.09)	1.21 (1.04, 1.41)
	Both	175, 2.86	61.13 (52.72, 70.90)	64.02 (54.51, 73.53)	0.90 (0.75, 1.07)	0.94 (0.78, 1.13)	1.16 (0.94, 1.43)
**VTE**	Unexposed	78, 9.34	8.35 (6.69, 10.43)	7.54 (5.84, 9.23)	1	1	0.72 (0.51, 1.02)
	Tamoxifen	58, 5.41	10.73 (8.30, 13.88)	10.36 (7.69, 13.03)	1.39 (0.98, 1.97)	1.39 (0.98, 1.98)	1
	AI	284, 33.99	8.36 (7.44, 9.39)	8.74 (7.71, 9.77)	1.17 (0.90, 1.52)	1.11 (0.84, 1.46)	0.80 (0.59, 1.07)
	Both	54, 4.31	12.54 (9.60, 16.37)	13.17 (9.65, 16.70)	1.67 (1.16, 2.41)	1.71 (1.18, 2.47)	1.23 (0.83, 1.81)
DVT	Unexposed	72, 9.36	7.69 (6.11, 9.69)	6.93 (5.31, 8.56)	1	1	0.71 (0.49, 1.02)
	Tamoxifen	54, 5.42	9.97 (7.63, 13.01)	9.62 (7.05, 12.19)	1.41 (0.98, 2.03)	1.42 (0.98, 2.04)	1
	AI	263, 34.05	7.72 (6.85, 8.72)	8.08 (7.09, 9.07)	1.18 (0.89, 1.56)	1.14 (0.86, 1.52)	0.81 (0.59, 1.09)
	Both	53, 4.31	12.29 (9.39, 16.09)	12.92 (9.43, 16.41)	1.79 (1.23, 2.60)	1.85 (1.26, 2.71)	1.31 (0.88, 1.94)
PE	Unexposed	-, -	0.93 (0.48, 1.78)	0.82 (0.27, 1.37)	1	1	0.95 (0.32, 2.87)
	Tamoxifen	-, -	0.91 (0.38, 2.18)	0.89 (0.11, 1.67)	1.02 (0.34, 3.06)	1.05 (0.35, 3.16)	1
	AI	29, 35.26	0.82 (0.57, 1.18)	0.87 (0.55, 1.19)	1.01 (0.47, 2.18)	0.80 (0.36, 1.79)	0.77 (0.29, 2.04)
	Both	-, -	0.22 (0.03, 1.58)	0.24 (-0.23, 0.70)	0.27 (0.03, 2.19)	0.26 (0.03, 2.05)	0.24 (0.03, 2.11)

*Events and follow-up supressed if number of events ≤ 11

**Adjusted for year of breast cancer diagnosis (2007-2013); age at index date (66-74, 75-84, 85+); race (White, Black Asian, Hispanic, Native American, other); SEER region (North East, South, North Central, West); breast cancer stage (1-3); breast cancer grade (1-3); time since index date (0-1yr, 1-3yrs, 3-5yrs, 5+yrs); current calendar year; use of taxanes, anthracyclines, trastuzumab, other systemic cancer treatments, statins, anti-hypertensive drugs, ACE inhibitors, calcium channel blockers, angiotensin receptor blockers; diagnosis of rheumatoid arthritis, chronic kidney disease, hypertension, diabetes, VTE, and non-venous CVD

***With reference category changed to ever tamoxifen

Matthews AA, *et al. Heart* 2021; 107:1327–1335. doi: 10.1136/heartjnl-2020-317510

Appendix 13 - Adjusted HRs for association between ever exposure to endocrine therapies and risk of CVD, stratified by age, time since index, and prior CVD in the UK

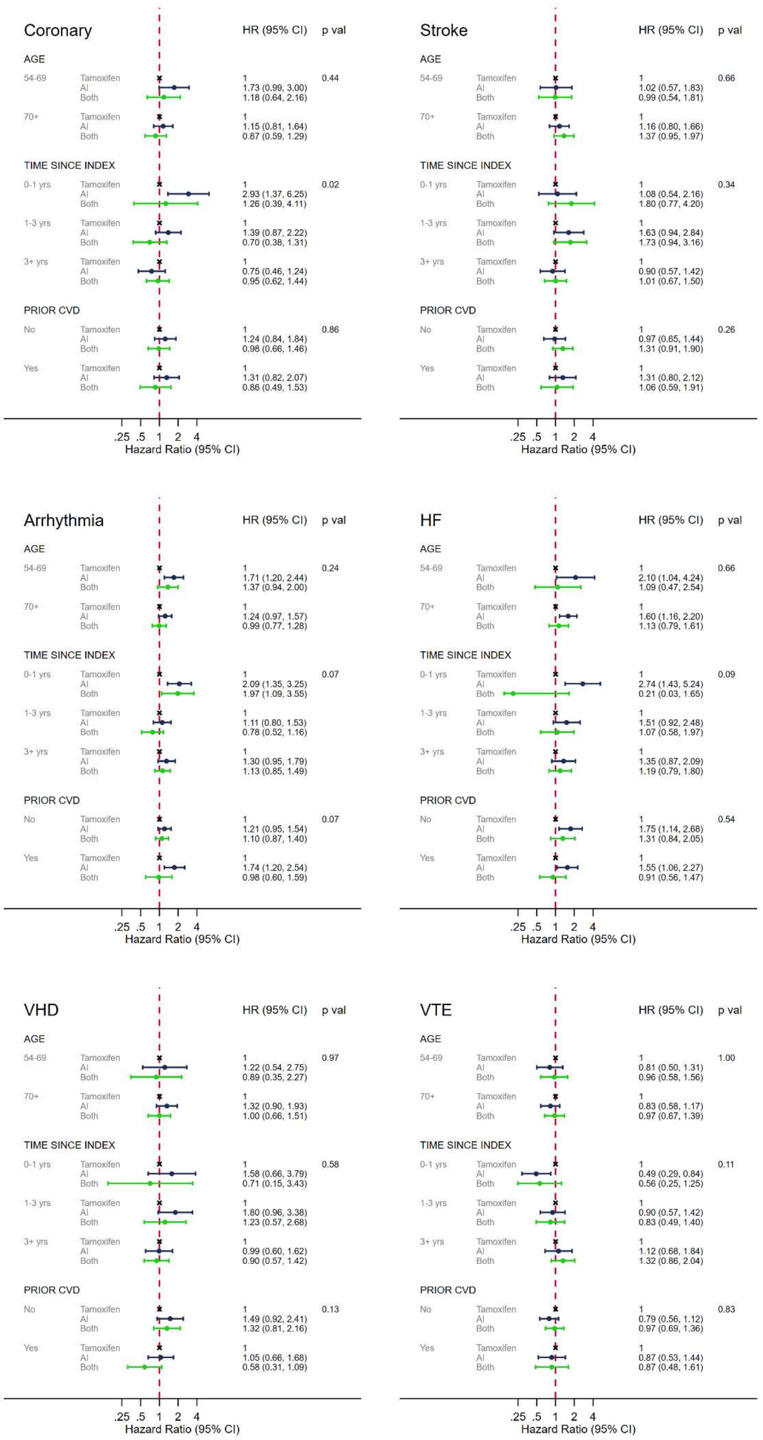

Matthews AA, *et al. Heart* 2021; 107:1327–1335. doi: 10.1136/heartjnl-2020-317510

Appendix 14 - Adjusted HRs for association between ever exposure to endocrine therapies and risk of CVD, stratified by age, time since index, and prior CVD in the US

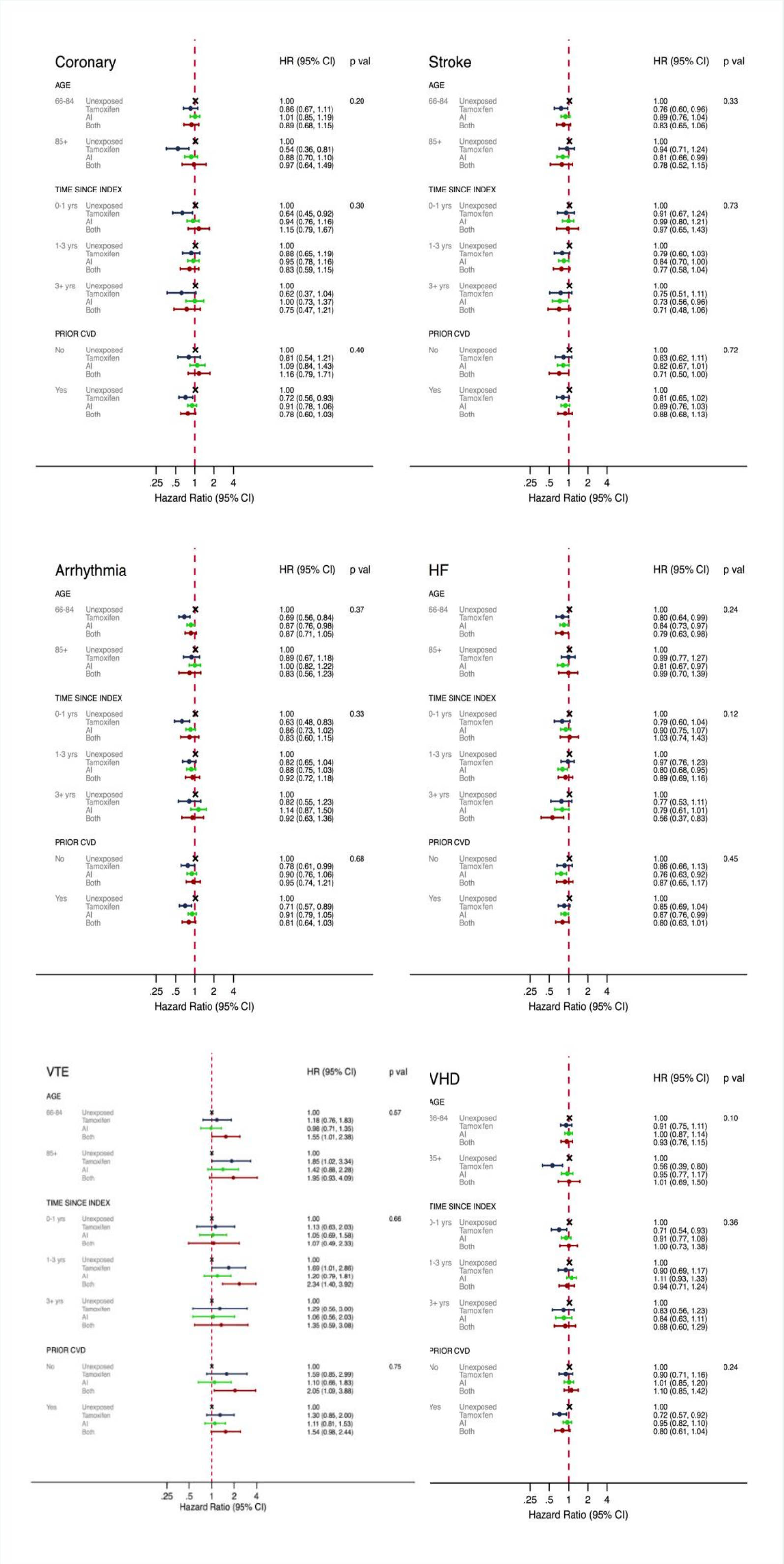

Matthews AA, *et al. Heart* 2021; 107:1327–1335. doi: 10.1136/heartjnl-2020-317510

Appendix 15 – Sensitivity analysis - Adjusted HRs for the association between ever AI compared to ever tamoxifen use and all CVD outcomes in modified analyses in both the UK and US

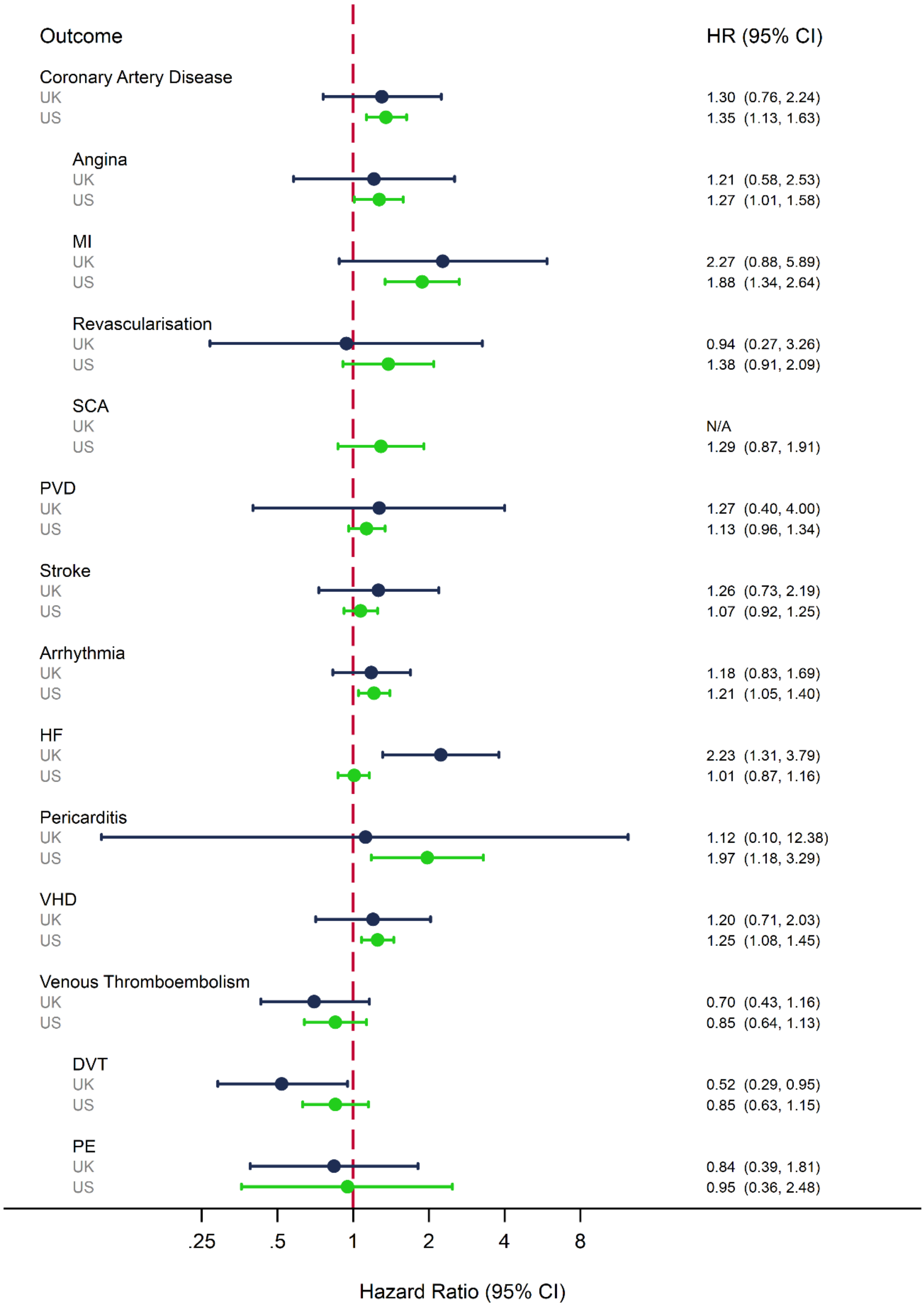

†Adjusted for: year of breast cancer; age at index date (66-74, 75-84, 85+); time since index date (0-1yr, 1-3yrs, 3-5yrs, 5+yrs); current calendar year; use of statins; use of ACE inhibitors; use of calcium channel blockers; use of angiotensin receptor blockers; rheumatoid arthritis; chronic kidney disease; diabetes; VTE; and non-venous CVD

Matthews AA, *et al. Heart* 2021; 107:1327–1335. doi: 10.1136/heartjnl-2020-317510

Appendix 16 – Sensitivity analysis - Quantitative bias analysis for potential unmeasured confounding results

The crude HR for the association between ever exposure to tamoxifen compared with those unexposed to any endocrine therapy and the risk of MI in the US was 0.44 (0.30, 0.63). The number of people with an MI event who were exposed to tamoxifen was 38, and there was 5410 person years of follow up in the ever tamoxifen group. The number of people with an MI event who were not exposed to any endocrine therapy was 153, and there was 9390 person years of follow up in the unexposed group. Based on the literature, we assumed the prevalence of the unmeasured confounder, frailty, in those that initiate endocrine therapy is 0.04 (this is unlikely to differ between those who initiate tamoxifen or AI), and the probability of frailty in those that do not initiate endocrine therapy is 0.1.[13] The relative risk between frailty and CVD is approximately 1.6 (this isn't the relative risk between frailty and MI specifically, but the RR specific to MI is unlikely greatly differ).[14] Given the above numbers, the crude HR would change from 0.44 to 0.45 if adjusted for frailty. These calculations can be checked by entering the above numbers into the spreadsheet at https://sites.google.com/site/biasanalysis/.

Furthermore, we can use the same methods to assume extreme values for the prevalence of any unmeasured confounder within levels of the exposure, and association between the confounder and MI. For example, assume the prevalence of the unmeasured confounder in those that initiate tamoxifen therapy was 0.1, the probability of the unmeasured confounder in those that do not initiate any endocrine therapy was 0.5, and the relative risk between the unmeasured confounder and MI was 3. The adjusted HR for the association between ever exposure to tamoxifen and risk of MI would then be 0.73. These proposed values are extreme, and unlikely to be true. However, they show that even in extreme cases, where one would assume large amounts of confounding, the association between ever exposure to endocrine therapy and risk of MI is not fully explained by the presence of information on the confounder. Unmeasured confounding is therefore unlikely to fully explain the observed association between ever exposure to tamoxifen and risk of MI.

Matthews AA, *et al. Heart* 2021; 107:1327–1335. doi: 10.1136/heartjnl-2020-317510

Appendix 17 - Adjusted HRs for the association between ever exposure to endocrine therapies and all CVD outcomes in the US, additionally adjusted for adjuvant radiotherapy

Outcome	Ever exposure	Adjusted HR (95% CI)*	Adjusted HR (95% CI)**
**Coronary Artery Disease**	Unexposed	1	1.33 (1.08, 1.64)
	Tamoxifen	0.75 (0.61, 0.93)	1
	AI	0.97 (0.84, 1.11)	1.29 (1.07, 1.56)
	Both	0.90 (0.72, 1.12)	1.19 (0.92, 1.55)
Angina	Unexposed	1	1.12 (0.87, 1.45)
	Tamoxifen	0.89 (0.69, 1.15)	1
	AI	1.06 (0.89, 1.27)	1.19 (0.95, 1.50)
	Both	0.96 (0.72, 1.26)	1.07 (0.78, 1.47)
MI	Unexposed		2.27 (1.57, 3.30)
	Tamoxifen	0.44 (0.30, 0.64)	1
	AI	0.80 (0.65, 0.98)	1.82 (1.28, 2.59)
	Both	0.68 (0.47, 0.97)	1.54 (0.98, 2.43)
Revascularisation	Unexposed	1	1.59 (0.99, 2.56)
	Tamoxifen	0.63 (0.39, 1.01)	1
	AI	0.92 (0.68, 1.24)	1.46 (0.95, 2.24)
	Both	0.99 (0.63, 1.56)	1.58 (0.91, 2.72)
SCA	Unexposed	1	1.47 (0.94, 2.29)
	Tamoxifen	0.68 (0.44, 1.06)	1
	AI	0.81 (0.61, 1.07)	1.18 (0.79, 1.78)
	Both	0.82 (0.51, 1.31)	1.21 (0.69, 2.10)
**PVD**	Unexposed	1	1.10 (0.90, 1.33)
	Tamoxifen	0.91 (0.75, 1.11)	1
	AI	1.00 (0.88, 1.15)	1.10 (0.93, 1.31)
	Both	0.93 (0.75, 1.15)	1.02 (0.80, 1.30)
**Stroke**	Unexposed	1	1.20 (1.00, 1.44)
	Tamoxifen	0.83 (0.70, 1.00)	1
	AI	0.88 (0.78, 1.00)	1.06 (0.90, 1.24)
	Both	0.82 (0.67, 1.01)	0.99 (0.79, 1.24)
**Arrhythmia**	Unexposed	1	1.33 (1.13, 1.57)
	Tamoxifen	0.75 (0.64, 0.88)	1
	AI	0.92 (0.82, 1.02)	1.22 (1.06, 1.41)
	Both	0.88 (0.74, 1.05)	1.18 (0.96, 1.44)
**HF**	Unexposed	1	1.14 (0.97, 1.34)
	Tamoxifen	0.87 (0.74, 1.03)	1
	AI	0.85 (0.76, 0.95)	0.97 (0.84, 1.12)
	Both	0.84 (0.70, 1.02)	0.96 (0.79, 1.19)
**Pericarditis**	Unexposed	1	2.68 (1.53, 4.68)
	Tamoxifen	0.37 (0.21, 0.65)	1
	AI	0.68 (0.51, 0.91)	1.81 (1.06, 3.09)
	Both	0.57 (0.34, 0.97)	1.53 (0.77, 3.05)
**VHD**	Unexposed	1	1.24 (1.05, 1.48)
	Tamoxifen	0.80 (0.68, 0.95)	1
	AI	0.97 (0.87, 1.09)	1.21 (1.04, 1.41)
	Both	0.93 (0.78, 1.12)	1.16 (0.94, 1.43)
**VTE**	Unexposed	1	0.72 (0.50, 1.02)
	Tamoxifen	1.40 (0.98, 1.99)	1
	AI	1.12 (0.85, 1.47)	0.80 (0.59, 1.07)
	Both	1.72 (1.19, 2.49)	1.23 (0.84, 1.81)
DVT	Unexposed	1	0.70 (0.49, 1.02)
	Tamoxifen	1.42 (0.99, 2.05)	1
	AI	1.15 (0.86, 1.53)	0.81 (0.59, 1.10)
	Both	1.86 (1.27, 2.73)	1.31 (0.88, 1.94)
PE	Unexposed	1	0.91 (0.30, 2.76)
	Tamoxifen	1.09 (0.36, 3.29)	1
	AI	0.85 (0.38, 1.90)	0.78 (0.29, 2.08)
	Both	0.27 (0.03, 2.14)	0.24 (0.03, 2.11)

*Adjusted for year of breast cancer diagnosis (2007-2013); age at index date (66-74, 75-84, 85+); race (White, Black Asian, Hispanic, Native American, other); SEER region (North East, South, North Central, West); breast cancer stage (1-3); breast cancer grade (1-3); time since index date (0-1yr, 1-3yrs, 3-5yrs, 5+yrs); current calendar year; use of taxanes, anthracyclines, trastuzumab, other systemic cancer treatments, statins, anti-hypertensive drugs, ACE inhibitors, calcium channel blockers, angiotensin receptor blockers; diagnosis of rheumatoid arthritis, chronic kidney disease, hypertension, diabetes, VTE, non-venous CVD, and adjuvant radiotherapy
**With reference category changed to ever tamoxifen

Appendix 18A – Sensitivity analysis - Crude and individually adjusted association between ever exposure to endocrine therapies and all CVD outcomes in the UK

Outcome	Exposure	Crude (with age as a timescale)	Year of cancer	Age	BMI	Alcohol	Smoking	Statins	ACEi	ARBs	Antiplatalets	CCBs
Coronary Artery Disease	Tamoxifen	1.00 (., .)	1.00 (., .)	1.00 (., .)	1.00 (., .)	1.00 (., .)	1.00 (., .)	1.00 (., .)	1.00 (., .)	1.00 (., .)	1.00 (., .)	1.00 (., .)
	AI	1.40 (1.06, 1.86)	1.49 (1.09, 2.02)	1.39 (1.05, 1.84)	1.40 (1.06, 1.85)	1.40 (1.06, 1.84)	1.41 (1.07, 1.86)	1.32 (0.99, 1.75)	1.38 (1.04, 1.83)	1.38 (1.04, 1.83)	1.34 (1.01, 1.77)	1.32 (0.99, 1.74)
	Both	0.95 (0.69, 1.31)	0.96 (0.69, 1.33)	0.95 (0.69, 1.31)	0.95 (0.69, 1.31)	0.95 (0.69, 1.32)	0.95 (0.69, 1.32)	0.94 (0.68, 1.29)	0.95 (0.69, 1.31)	0.95 (0.69, 1.31)	0.95 (0.68, 1.31)	0.94 (0.68, 1.30)
Angina	Tamoxifen	1.00 (., .)	1.00 (., .)	1.00 (., .)	1.00 (., .)	1.00 (., .)	1.00 (., .)	1.00 (., .)	1.00 (., .)	1.00 (., .)	1.00 (., .)	1.00 (., .)
	AI	1.43 (1.00, 2.05)	1.64 (1.11, 2.44)	1.40 (0.98, 2.01)	1.41 (0.98, 2.02)	1.44 (1.01, 2.06)	1.41 (0.99, 2.02)	1.26 (0.88, 1.81)	1.36 (0.95, 1.96)	1.39 (0.97, 2.00)	1.33 (0.93, 1.90)	1.33 (0.93, 1.90)
	Both	0.70 (0.44, 1.10)	0.72 (0.45, 1.13)	0.71 (0.45, 1.13)	0.70 (0.44, 1.10)	0.70 (0.44, 1.11)	0.70 (0.44, 1.10)	0.69 (0.43, 1.08)	0.70 (0.44, 1.11)	0.71 (0.45, 1.11)	0.70 (0.44, 1.10)	0.70 (0.44, 1.10)
MI	Tamoxifen	1.00 (., .)	1.00 (., .)	1.00 (., .)	1.00 (., .)	1.00 (., .)	1.00 (., .)	1.00 (., .)	1.00 (., .)	1.00 (., .)	1.00 (., .)	1.00 (., .)
	AI	1.73 (1.12, 2.68)	1.73 (1.07, 2.77)	1.70 (1.10, 2.64)	1.76 (1.13, 2.72)	1.71 (1.10, 2.64)	1.73 (1.12, 2.69)	1.62 (1.04, 2.52)	1.71 (1.10, 2.66)	1.72 (1.11, 2.67)	1.66 (1.07, 2.57)	1.63 (1.05, 2.53)
	Both	1.62 (1.01, 2.60)	1.60 (0.99, 2.57)	1.62 (1.01, 2.60)	1.63 (1.01, 2.61)	1.63 (1.02, 2.62)	1.64 (1.02, 2.63)	1.60 (1.00, 2.56)	1.62 (1.01, 2.60)	1.62 (1.01, 2.60)	1.63 (1.01, 2.61)	1.62 (1.01, 2.59)
Revascularisation	Tamoxifen	1.00 (., .)	1.00 (., .)	1.00 (., .)	1.00 (., .)	1.00 (., .)	1.00 (., .)	1.00 (., .)	1.00 (., .)	1.00 (., .)	1.00 (., .)	1.00 (., .)
	AI	1.36 (0.69, 2.68)	1.86 (0.87, 3.95)	1.39 (0.70, 2.75)	1.31 (0.66, 2.59)	1.41 (0.71, 2.77)	1.37 (0.70, 2.71)	1.24 (0.62, 2.47)	1.28 (0.65, 2.54)	1.34 (0.68, 2.65)	1.30 (0.66, 2.57)	1.27 (0.64, 2.52)
	Both	0.99 (0.46, 2.11)	1.03 (0.48, 2.22)	0.97 (0.45, 2.09)	0.99 (0.46, 2.11)	0.98 (0.46, 2.10)	0.99 (0.46, 2.12)	0.98 (0.46, 2.11)	1.00 (0.47, 2.14)	0.99 (0.46, 2.12)	0.99 (0.46, 2.12)	0.99 (0.46, 2.11)
SCA	Tamoxifen	1.00 (., .)	1.00 (., .)	1.00 (., .)	1.00 (., .)	1.00 (., .)	1.00 (., .)	1.00 (., .)	1.00 (., .)	1.00 (., .)	1.00 (., .)	1.00 (., .)
	AI	1.87 (0.83, 4.22)	1.72 (0.70, 4.23)	1.96 (0.87, 4.45)	1.85 (0.82, 4.18)	1.82 (0.81, 4.11)	1.85 (0.82, 4.16)	1.89 (0.83, 4.31)	1.89 (0.84, 4.26)	1.91 (0.85, 4.31)	1.84 (0.82, 4.16)	1.79 (0.79, 4.05)
	Both	0.73 (0.24, 2.19)	0.74 (0.25, 2.22)	0.69 (0.23, 2.08)	0.73 (0.24, 2.19)	0.74 (0.25, 2.22)	0.73 (0.24, 2.18)	0.73 (0.24, 2.20)	0.73 (0.24, 2.19)	0.73 (0.24, 2.18)	0.74 (0.25, 2.20)	0.73 (0.24, 2.19)
PVD	Tamoxifen	1.00 (., .)	1.00 (., .)	1.00 (., .)	1.00 (., .)	1.00 (., .)	1.00 (., .)	1.00 (., .)	1.00 (., .)	1.00 (., .)	1.00 (., .)	1.00 (., .)
	AI	1.25 (0.77, 2.01)	1.47 (0.86, 2.49)	1.28 (0.79, 2.08)	1.23 (0.76, 1.99)	1.25 (0.77, 2.03)	1.23 (0.76, 1.99)	1.17 (0.72, 1.90)	1.18 (0.73, 1.90)	1.23 (0.76, 2.00)	1.23 (0.76, 1.99)	1.17 (0.72, 1.89)
	Both	0.81 (0.45, 1.43)	0.81 (0.45, 1.44)	0.78 (0.44, 1.39)	0.80 (0.45, 1.43)	0.81 (0.45, 1.44)	0.80 (0.45, 1.43)	0.80 (0.45, 1.42)	0.82 (0.46, 1.45)	0.81 (0.46, 1.44)	0.81 (0.45, 1.44)	0.81 (0.46, 1.44)
Stroke	Tamoxifen	1.00 (., .)	1.00 (., .)	1.00 (., .)	1.00 (., .)	1.00 (., .)	1.00 (., .)	1.00 (., .)	1.00 (., .)	1.00 (., .)	1.00 (., .)	1.00 (., .)
	AI	1.22 (0.91, 1.63)	1.18 (0.86, 1.62)	1.22 (0.92, 1.64)	1.22 (0.91, 1.64)	1.22 (0.91, 1.63)	1.22 (0.91, 1.62)	1.16 (0.86, 1.55)	1.19 (0.89, 1.60)	1.21 (0.91, 1.62)	1.19 (0.89, 1.59)	1.18 (0.88, 1.58)
	Both	1.33 (0.98, 1.80)	1.31 (0.97, 1.79)	1.31 (0.96, 1.78)	1.33 (0.98, 1.80)	1.33 (0.98, 1.80)	1.33 (0.98, 1.80)	1.31 (0.97, 1.78)	1.33 (0.98, 1.80)	1.33 (0.98, 1.80)	1.33 (0.98, 1.81)	1.33 (0.98, 1.80)
Arrhythmia	Tamoxifen	1.00 (., .)	1.00 (., .)	1.00 (., .)	1.00 (., .)	1.00 (., .)	1.00 (., .)	1.00 (., .)	1.00 (., .)	1.00 (., .)	1.00 (., .)	1.00 (., .)
	AI	1.37 (1.14, 1.64)	1.45 (1.18, 1.78)	1.38 (1.15, 1.66)	1.34 (1.12, 1.62)	1.36 (1.13, 1.64)	1.35 (1.12, 1.63)	1.31 (1.08, 1.58)	1.32 (1.10, 1.59)	1.35 (1.12, 1.63)	1.34 (1.11, 1.61)	1.32 (1.10, 1.59)
	Both	1.07 (0.87, 1.32)	1.09 (0.88, 1.35)	1.06 (0.86, 1.31)	1.07 (0.87, 1.32)	1.08 (0.88, 1.33)	1.07 (0.87, 1.31)	1.07 (0.87, 1.31)	1.07 (0.87, 1.32)	1.07 (0.87, 1.32)	1.07 (0.87, 1.32)	1.07 (0.87, 1.32)
HF	Tamoxifen	1.00 (., .)	1.00 (., .)	1.00 (., .)	1.00 (., .)	1.00 (., .)	1.00 (., .)	1.00 (., .)	1.00 (., .)	1.00 (., .)	1.00 (., .)	1.00 (., .)
	AI	1.87 (1.43, 2.45)	1.94 (1.44, 2.60)	1.89 (1.44, 2.47)	1.80 (1.38, 2.36)	1.87 (1.43, 2.44)	1.83 (1.40, 2.40)	1.69 (1.29, 2.22)	1.74 (1.33, 2.28)	1.83 (1.40, 2.40)	1.75 (1.34, 2.29)	1.78 (1.36, 2.33)
	Both	1.12 (0.81, 1.55)	1.11 (0.81, 1.54)	1.11 (0.81, 1.54)	1.12 (0.81, 1.54)	1.13 (0.82, 1.57)	1.11 (0.81, 1.54)	1.10 (0.80, 1.52)	1.12 (0.81, 1.55)	1.14 (0.82, 1.57)	1.13 (0.82, 1.56)	1.12 (0.81, 1.54)
Pericarditis	Tamoxifen	1.00 (., .)	1.00 (., .)	1.00 (., .)	1.00 (., .)	1.00 (., .)	1.00 (., .)	1.00 (., .)	1.00 (., .)	1.00 (., .)	1.00 (., .)	1.00 (., .)
	AI	3.96 (1.12, 14.03)	3.40 (0.91, 12.77)	4.09 (1.15, 14.57)	3.84 (1.08, 13.65)	4.15 (1.17, 14.71)	3.99 (1.12, 14.16)	4.06 (1.14, 14.48)	3.69 (1.04, 13.13)	3.89 (1.10, 13.82)	3.91 (1.10, 13.89)	3.94 (1.11, 14.01)
	Both	4.29 (1.18, 15.61)	3.82 (1.04, 13.96)	4.17 (1.14, 15.26)	4.29 (1.18, 15.59)	4.21 (1.16, 15.32)	4.30 (1.18, 15.63)	4.30 (1.18, 15.63)	4.44 (1.22, 16.17)	4.33 (1.19, 15.77)	4.31 (1.19, 15.70)	4.30 (1.18, 15.63)
VHD	Tamoxifen	1.00 (., .)	1.00 (., .)	1.00 (., .)	1.00 (., .)	1.00 (., .)	1.00 (., .)	1.00 (., .)	1.00 (., .)	1.00 (., .)	1.00 (., .)	1.00 (., .)
	AI	1.58 (1.15, 2.17)	1.43 (1.01, 2.03)	1.61 (1.17, 2.21)	1.56 (1.13, 2.15)	1.57 (1.14, 2.15)	1.57 (1.14, 2.16)	1.45 (1.05, 2.01)	1.50 (1.09, 2.07)	1.57 (1.14, 2.16)	1.52 (1.11, 2.10)	1.52 (1.10, 2.10)
	Both	1.08 (0.74, 1.57)	1.10 (0.75, 1.60)	1.06 (0.73, 1.54)	1.07 (0.74, 1.56)	1.09 (0.75, 1.58)	1.07 (0.74, 1.56)	1.06 (0.73, 1.55)	1.09 (0.75, 1.58)	1.08 (0.74, 1.57)	1.08 (0.74, 1.57)	1.07 (0.74, 1.56)
VTE	Tamoxifen	1.00 (., .)	1.00 (., .)	1.00 (., .)	1.00 (., .)	1.00 (., .)	1.00 (., .)	1.00 (., .)	1.00 (., .)	1.00 (., .)	1.00 (., .)	1.00 (., .)
	AI	0.93 (0.71, 1.21)	0.86 (0.64, 1.14)	0.92 (0.71, 1.20)	0.90 (0.69, 1.17)	0.92 (0.71, 1.20)	0.91 (0.69, 1.18)	0.89 (0.68, 1.16)	0.91 (0.69, 1.18)	0.93 (0.71, 1.21)	0.92 (0.71, 1.20)	0.91 (0.70, 1.19)
	Both	0.91 (0.69, 1.22)	0.90 (0.67, 1.20)	0.91 (0.68, 1.22)	0.91 (0.69, 1.22)	0.92 (0.69, 1.22)	0.90 (0.68, 1.20)	0.91 (0.68, 1.21)	0.92 (0.69, 1.22)	0.91 (0.69, 1.22)	0.91 (0.69, 1.22)	0.91 (0.69, 1.22)
DVT	Tamoxifen	1.00 (., .)	1.00 (., .)	1.00 (., .)	1.00 (., .)	1.00 (., .)	1.00 (., .)	1.00 (., .)	1.00 (., .)	1.00 (., .)	1.00 (., .)	1.00 (., .)
	AI	0.69 (0.48, 0.98)	0.66 (0.45, 0.96)	0.68 (0.48, 0.97)	0.66 (0.46, 0.94)	0.68 (0.48, 0.97)	0.67 (0.47, 0.95)	0.65 (0.46, 0.94)	0.68 (0.47, 0.96)	0.69 (0.48, 0.98)	0.68 (0.48, 0.97)	0.68 (0.48, 0.97)
	Both	0.98 (0.69, 1.38)	0.96 (0.68, 1.36)	0.99 (0.70, 1.40)	0.97 (0.69, 1.37)	0.98 (0.69, 1.39)	0.96 (0.68, 1.35)	0.97 (0.69, 1.37)	0.98 (0.69, 1.38)	0.98 (0.69, 1.38)	0.98 (0.69, 1.38)	0.98 (0.69, 1.38)
PE	Tamoxifen	1.00 (., .)	1.00 (., .)	1.00 (., .)	1.00 (., .)	1.00 (., .)	1.00 (., .)	1.00 (., .)	1.00 (., .)	1.00 (., .)	1.00 (., .)	1.00 (., .)
	AI	1.39 (0.95, 2.04)	1.35 (0.89, 2.04)	1.40 (0.96, 2.05)	1.34 (0.92, 1.97)	1.38 (0.94, 2.02)	1.37 (0.94, 2.00)	1.38 (0.94, 2.02)	1.37 (0.93, 2.00)	1.39 (0.95, 2.03)	1.39 (0.95, 2.03)	1.36 (0.93, 1.99)
	Both	0.80 (0.51, 1.28)	0.79 (0.50, 1.26)	0.79 (0.50, 1.27)	0.80 (0.50, 1.28)	0.81 (0.51, 1.29)	0.80 (0.50, 1.28)	0.80 (0.50, 1.28)	0.81 (0.51, 1.29)	0.80 (0.51, 1.28)	0.80 (0.51, 1.28)	0.81 (0.51, 1.28)

Matthews AA, *et al. Heart* 2021; 107:1327–1335. doi: 10.1136/heartjnl-2020-317510

Appendix 18B – Sensitivity analysis - Crude and individually adjusted association between ever exposure to endocrine therapies and all CVD outcomes in the UK, continued

Outcome	Exposure	Crude (with age as a timescale)	Diabetes	RA	CKD	Systolic BP	Diastolic BP	IMD	Time since index	Current year	Prior VTE	Prior non-venous CVD
Coronary Artery Disease	Tamoxifen	1.00 (., .)	1.00 (., .)	1.00 (., .)	1.00 (., .)	1.00 (., .)	1.00 (., .)	1.00 (., .)	1.00 (., .)	1.00 (., .)	1.00 (., .)	1.00 (., .)
	AI	1.40 (1.06, 1.86)	1.38 (1.04, 1.83)	1.40 (1.06, 1.86)	1.40 (1.06, 1.85)	1.40 (1.06, 1.85)	1.41 (1.07, 1.86)	1.41 (1.07, 1.87)	1.41 (1.07, 1.87)	1.47 (1.10, 1.96)	1.38 (1.04, 1.82)	1.28 (0.96, 1.69)
	Both	0.95 (0.69, 1.31)	0.95 (0.69, 1.31)	0.95 (0.69, 1.31)	0.95 (0.69, 1.31)	0.95 (0.69, 1.31)	0.95 (0.69, 1.31)	0.95 (0.69, 1.32)	0.94 (0.68, 1.30)	0.97 (0.70, 1.35)	0.94 (0.68, 1.30)	0.95 (0.69, 1.31)
Angina	Tamoxifen	1.00 (., .)	1.00 (., .)	1.00 (., .)	1.00 (., .)	1.00 (., .)	1.00 (., .)	1.00 (., .)	1.00 (., .)	1.00 (., .)	1.00 (., .)	1.00 (., .)
	AI	1.43 (1.00, 2.05)	1.40 (0.98, 2.00)	1.43 (1.00, 2.05)	1.43 (1.00, 2.04)	1.42 (0.99, 2.04)	1.43 (1.00, 2.05)	1.45 (1.01, 2.07)	1.43 (0.99, 2.05)	1.58 (1.09, 2.29)	1.39 (0.97, 1.99)	1.27 (0.88, 1.82)
	Both	0.70 (0.44, 1.10)	0.70 (0.44, 1.11)	0.70 (0.44, 1.10)	0.70 (0.44, 1.10)	0.70 (0.44, 1.10)	0.70 (0.44, 1.10)	0.71 (0.45, 1.12)	0.69 (0.43, 1.09)	0.73 (0.46, 1.15)	0.69 (0.43, 1.09)	0.70 (0.44, 1.11)
MI	Tamoxifen	1.00 (., .)	1.00 (., .)	1.00 (., .)	1.00 (., .)	1.00 (., .)	1.00 (., .)	1.00 (., .)	1.00 (., .)	1.00 (., .)	1.00 (., .)	1.00 (., .)
	AI	1.73 (1.12, 2.68)	1.69 (1.09, 2.63)	1.73 (1.12, 2.68)	1.71 (1.11, 2.66)	1.74 (1.12, 2.70)	1.73 (1.12, 2.68)	1.74 (1.13, 2.70)	1.72 (1.11, 2.68)	1.77 (1.13, 2.77)	1.72 (1.11, 2.67)	1.59 (1.02, 2.47)
	Both	1.62 (1.01, 2.60)	1.62 (1.01, 2.60)	1.62 (1.01, 2.60)	1.62 (1.01, 2.60)	1.62 (1.01, 2.60)	1.62 (1.01, 2.59)	1.63 (1.02, 2.62)	1.62 (1.00, 2.60)	1.65 (1.02, 2.66)	1.62 (1.01, 2.59)	1.67 (1.04, 2.68)
Revascularisation	Tamoxifen	1.00 (., .)	1.00 (., .)	1.00 (., .)	1.00 (., .)	1.00 (., .)	1.00 (., .)	1.00 (., .)	1.00 (., .)	1.00 (., .)	1.00 (., .)	1.00 (., .)
	AI	1.36 (0.69, 2.68)	1.30 (0.66, 2.57)	1.36 (0.69, 2.68)	1.36 (0.69, 2.68)	1.39 (0.71, 2.75)	1.37 (0.70, 2.71)	1.35 (0.69, 2.67)	1.48 (0.74, 2.93)	1.51 (0.74, 3.08)	1.44 (0.73, 2.84)	1.22 (0.62, 2.42)
	Both	0.99 (0.46, 2.11)	0.99 (0.46, 2.11)	0.99 (0.46, 2.11)	0.99 (0.46, 2.11)	1.00 (0.47, 2.14)	0.98 (0.46, 2.11)	0.98 (0.46, 2.10)	0.89 (0.41, 1.91)	1.07 (0.49, 2.31)	1.02 (0.48, 2.18)	1.00 (0.47, 2.13)
SCA	Tamoxifen	1.00 (., .)	1.00 (., .)	1.00 (., .)	1.00 (., .)	1.00 (., .)	1.00 (., .)	1.00 (., .)	1.00 (., .)	1.00 (., .)	1.00 (., .)	1.00 (., .)
	AI	1.87 (0.83, 4.22)	1.92 (0.85, 4.32)	1.89 (0.84, 4.26)	1.87 (0.83, 4.22)	1.78 (0.79, 4.02)	1.88 (0.83, 4.24)	1.90 (0.84, 4.28)	1.96 (0.86, 4.48)	1.66 (0.72, 3.84)	1.92 (0.85, 4.34)	1.82 (0.81, 4.13)
	Both	0.73 (0.24, 2.19)	0.73 (0.24, 2.17)	0.73 (0.24, 2.19)	0.73 (0.24, 2.19)	0.72 (0.24, 2.15)	0.73 (0.24, 2.18)	0.75 (0.25, 2.25)	0.71 (0.24, 2.15)	0.69 (0.23, 2.09)	0.74 (0.25, 2.22)	0.73 (0.25, 2.20)
PVD	Tamoxifen	1.00 (., .)	1.00 (., .)	1.00 (., .)	1.00 (., .)	1.00 (., .)	1.00 (., .)	1.00 (., .)	1.00 (., .)	1.00 (., .)	1.00 (., .)	1.00 (., .)
	AI	1.25 (0.77, 2.01)	1.18 (0.73, 1.91)	1.25 (0.77, 2.01)	1.24 (0.77, 2.01)	1.26 (0.78, 2.04)	1.25 (0.77, 2.02)	1.26 (0.78, 2.03)	1.25 (0.77, 2.03)	1.44 (0.87, 2.39)	1.23 (0.76, 2.00)	1.19 (0.73, 1.93)
	Both	0.81 (0.45, 1.43)	0.81 (0.45, 1.44)	0.81 (0.46, 1.44)	0.81 (0.46, 1.44)	0.81 (0.46, 1.44)	0.81 (0.45, 1.43)	0.81 (0.46, 1.45)	0.82 (0.46, 1.46)	0.88 (0.49, 1.59)	0.80 (0.45, 1.43)	0.82 (0.46, 1.45)
Stroke	Tamoxifen	1.00 (., .)	1.00 (., .)	1.00 (., .)	1.00 (., .)	1.00 (., .)	1.00 (., .)	1.00 (., .)	1.00 (., .)	1.00 (., .)	1.00 (., .)	1.00 (., .)
	AI	1.22 (0.91, 1.63)	1.20 (0.90, 1.60)	1.22 (0.91, 1.63)	1.22 (0.91, 1.63)	1.21 (0.91, 1.62)	1.23 (0.92, 1.64)	1.22 (0.91, 1.63)	1.25 (0.93, 1.67)	1.16 (0.86, 1.57)	1.21 (0.91, 1.63)	1.18 (0.88, 1.57)
	Both	1.33 (0.98, 1.80)	1.33 (0.98, 1.81)	1.33 (0.98, 1.80)	1.32 (0.98, 1.80)	1.32 (0.97, 1.79)	1.32 (0.97, 1.79)	1.33 (0.98, 1.80)	1.29 (0.95, 1.75)	1.28 (0.94, 1.74)	1.32 (0.97, 1.80)	1.34 (0.99, 1.83)
Arrhythmia	Tamoxifen	1.00 (., .)	1.00 (., .)	1.00 (., .)	1.00 (., .)	1.00 (., .)	1.00 (., .)	1.00 (., .)	1.00 (., .)	1.00 (., .)	1.00 (., .)	1.00 (., .)
	AI	1.37 (1.14, 1.64)	1.36 (1.13, 1.63)	1.37 (1.14, 1.65)	1.36 (1.13, 1.64)	1.38 (1.14, 1.66)	1.37 (1.14, 1.64)	1.37 (1.14, 1.65)	1.38 (1.15, 1.67)	1.38 (1.14, 1.67)	1.35 (1.12, 1.63)	1.32 (1.10, 1.59)
	Both	1.07 (0.87, 1.32)	1.07 (0.87, 1.32)	1.07 (0.87, 1.32)	1.07 (0.87, 1.32)	1.07 (0.87, 1.32)	1.07 (0.87, 1.32)	1.08 (0.87, 1.33)	1.06 (0.86, 1.30)	1.07 (0.87, 1.32)	1.07 (0.86, 1.31)	1.10 (0.89, 1.35)
HF	Tamoxifen	1.00 (., .)	1.00 (., .)	1.00 (., .)	1.00 (., .)	1.00 (., .)	1.00 (., .)	1.00 (., .)	1.00 (., .)	1.00 (., .)	1.00 (., .)	1.00 (., .)
	AI	1.87 (1.43, 2.45)	1.83 (1.40, 2.40)	1.87 (1.43, 2.44)	1.84 (1.41, 2.41)	1.89 (1.44, 2.47)	1.85 (1.41, 2.42)	1.89 (1.44, 2.47)	1.90 (1.45, 2.50)	1.85 (1.40, 2.44)	1.83 (1.39, 2.40)	1.70 (1.30, 2.22)
	Both	1.12 (0.81, 1.55)	1.13 (0.82, 1.56)	1.12 (0.82, 1.55)	1.13 (0.82, 1.56)	1.13 (0.82, 1.56)	1.12 (0.81, 1.55)	1.14 (0.82, 1.57)	1.12 (0.81, 1.55)	1.11 (0.80, 1.54)	1.11 (0.81, 1.54)	1.15 (0.84, 1.59)
Pericarditis	Tamoxifen	1.00 (., .)	1.00 (., .)	1.00 (., .)	1.00 (., .)	1.00 (., .)	1.00 (., .)	1.00 (., .)	1.00 (., .)	1.00 (., .)	1.00 (., .)	1.00 (., .)
	AI	3.96 (1.12, 14.03)	3.65 (1.02, 12.97)	3.96 (1.12, 14.06)	3.94 (1.11, 13.98)	3.92 (1.10, 13.92)	3.91 (1.10, 13.90)	3.88 (1.09, 13.76)	4.31 (1.20, 15.40)	3.00 (0.83, 10.84)	4.18 (1.18, 14.84)	3.87 (1.09, 13.78)
	Both	4.29 (1.18, 15.61)	4.35 (1.20, 15.85)	4.32 (1.19, 15.73)	4.29 (1.18, 15.61)	4.30 (1.18, 15.64)	4.28 (1.18, 15.56)	4.25 (1.17, 15.47)	4.03 (1.09, 14.86)	3.62 (0.99, 13.26)	4.40 (1.21, 16.01)	4.31 (1.18, 15.68)
VHD	Tamoxifen	1.00 (., .)	1.00 (., .)	1.00 (., .)	1.00 (., .)	1.00 (., .)	1.00 (., .)	1.00 (., .)	1.00 (., .)	1.00 (., .)	1.00 (., .)	1.00 (., .)
	AI	1.58 (1.15, 2.17)	1.57 (1.14, 2.16)	1.58 (1.15, 2.18)	1.57 (1.14, 2.16)	1.58 (1.15, 2.18)	1.56 (1.14, 2.15)	1.58 (1.15, 2.18)	1.66 (1.20, 2.29)	1.33 (0.96, 1.84)	1.57 (1.14, 2.16)	1.49 (1.08, 2.05)
	Both	1.08 (0.74, 1.57)	1.08 (0.74, 1.57)	1.08 (0.74, 1.57)	1.08 (0.74, 1.57)	1.08 (0.74, 1.57)	1.08 (0.74, 1.57)	1.07 (0.74, 1.56)	1.02 (0.70, 1.49)	0.96 (0.66, 1.41)	1.07 (0.74, 1.56)	1.10 (0.75, 1.60)
VTE	Tamoxifen	1.00 (., .)	1.00 (., .)	1.00 (., .)	1.00 (., .)	1.00 (., .)	1.00 (., .)	1.00 (., .)	1.00 (., .)	1.00 (., .)	1.00 (., .)	1.00 (., .)
	AI	0.93 (0.71, 1.21)	0.92 (0.70, 1.20)	0.93 (0.71, 1.21)	0.92 (0.71, 1.21)	0.93 (0.71, 1.21)	0.94 (0.72, 1.22)	0.93 (0.71, 1.22)	0.88 (0.67, 1.15)	0.98 (0.74, 1.29)	0.93 (0.71, 1.21)	0.92 (0.70, 1.20)
	Both	0.91 (0.69, 1.22)	0.92 (0.69, 1.22)	0.91 (0.69, 1.22)	0.92 (0.69, 1.22)	0.91 (0.69, 1.22)	0.91 (0.69, 1.22)	0.92 (0.69, 1.22)	0.97 (0.73, 1.30)	0.95 (0.71, 1.28)	0.91 (0.69, 1.22)	0.91 (0.69, 1.22)
DVT	Tamoxifen	1.00 (., .)	1.00 (., .)	1.00 (., .)	1.00 (., .)	1.00 (., .)	1.00 (., .)	1.00 (., .)	1.00 (., .)	1.00 (., .)	1.00 (., .)	1.00 (., .)
	AI	0.69 (0.48, 0.98)	0.68 (0.48, 0.97)	0.69 (0.48, 0.98)	0.69 (0.48, 0.98)	0.68 (0.48, 0.97)	0.69 (0.48, 0.98)	0.69 (0.48, 0.98)	0.65 (0.45, 0.92)	0.75 (0.52, 1.09)	0.68 (0.48, 0.97)	0.69 (0.48, 0.98)
	Both	0.98 (0.69, 1.38)	0.98 (0.69, 1.38)	0.98 (0.69, 1.38)	0.98 (0.69, 1.38)	0.97 (0.69, 1.38)	0.98 (0.69, 1.38)	0.98 (0.69, 1.38)	1.07 (0.75, 1.51)	1.05 (0.74, 1.50)	0.97 (0.69, 1.37)	0.98 (0.69, 1.38)
PE	Tamoxifen	1.00 (., .)	1.00 (., .)	1.00 (., .)	1.00 (., .)	1.00 (., .)	1.00 (., .)	1.00 (., .)	1.00 (., .)	1.00 (., .)	1.00 (., .)	1.00 (., .)
	AI	1.39 (0.95, 2.04)	1.38 (0.94, 2.02)	1.39 (0.95, 2.03)	1.39 (0.95, 2.03)	1.41 (0.97, 2.07)	1.41 (0.97, 2.07)	1.40 (0.96, 2.05)	1.35 (0.92, 1.98)	1.44 (0.97, 2.14)	1.31 (0.89, 1.92)	1.38 (0.94, 2.02)
	Both	0.80 (0.51, 1.28)	0.81 (0.51, 1.28)	0.80 (0.50, 1.28)	0.81 (0.51, 1.29)	0.81 (0.51, 1.29)	0.80 (0.50, 1.28)	0.80 (0.51, 1.28)	0.82 (0.51, 1.32)	0.81 (0.51, 1.30)	0.78 (0.49, 1.24)	0.80 (0.51, 1.28)

Appendix 19A – Sensitivity analysis - Crude and individually adjusted association between ever exposure to endocrine therapies and all CVD outcomes in the US

Outcome	Exposure	Crude (with age as a timescale)	Age	Race	SEER region	Breast cancer stage	Breast cancer grade	Time since index	Taxanes	Anthracyclines	Trastuzumab	Current year	Other cancer therapies
Coronary Artery Disease	Unexposed	1.00 (., .)	1.00 (., .)	1.00 (., .)	1.00 (., .)	1.00 (., .)	1.00 (., .)	1.00 (., .)	1.00 (., .)	1.00 (., .)	1.00 (., .)	1.00 (., .)	1.00 (., .)
	Tamoxifen	0.73 (0.60, 0.90)	0.74 (0.60, 0.90)	0.73 (0.60, 0.90)	0.73 (0.60, 0.90)	0.72 (0.59, 0.89)	0.73 (0.60, 0.90)	0.74 (0.60, 0.91)	0.73 (0.60, 0.90)	0.73 (0.60, 0.90)	0.73 (0.60, 0.90)	0.74 (0.60, 0.90)	0.73 (0.60, 0.90)
	AI	1.04 (0.91, 1.18)	1.04 (0.92, 1.19)	1.03 (0.91, 1.17)	1.02 (0.89, 1.16)	1.00 (0.88, 1.14)	1.00 (0.87, 1.14)	1.04 (0.92, 1.19)	1.04 (0.91, 1.18)	1.04 (0.91, 1.18)	1.04 (0.91, 1.18)	1.06 (0.93, 1.20)	1.03 (0.91, 1.17)
	Both	0.89 (0.72, 1.11)	0.90 (0.73, 1.11)	0.91 (0.73, 1.12)	0.88 (0.71, 1.09)	0.87 (0.70, 1.07)	0.85 (0.69, 1.06)	0.91 (0.74, 1.13)	0.89 (0.72, 1.10)	0.89 (0.72, 1.10)	0.89 (0.72, 1.11)	0.91 (0.74, 1.13)	0.89 (0.72, 1.10)
Angina	Unexposed	1.00 (., .)	1.00 (., .)	1.00 (., .)	1.00 (., .)	1.00 (., .)	1.00 (., .)	1.00 (., .)	1.00 (., .)	1.00 (., .)	1.00 (., .)	1.00 (., .)	1.00 (., .)
	Tamoxifen	0.82 (0.63, 1.05)	0.82 (0.64, 1.06)	0.83 (0.64, 1.07)	0.83 (0.64, 1.07)	0.82 (0.63, 1.05)	0.84 (0.65, 1.08)	0.83 (0.64, 1.07)	0.82 (0.63, 1.05)	0.82 (0.63, 1.05)	0.82 (0.63, 1.05)	0.82 (0.63, 1.06)	0.82 (0.63, 1.05)
	AI	1.08 (0.92, 1.27)	1.08 (0.92, 1.28)	1.08 (0.91, 1.27)	1.06 (0.90, 1.25)	1.08 (0.91, 1.27)	1.04 (0.88, 1.23)	1.09 (0.92, 1.28)	1.08 (0.91, 1.27)	1.08 (0.92, 1.27)	1.08 (0.92, 1.27)	1.11 (0.94, 1.31)	1.07 (0.91, 1.27)
	Both	0.88 (0.68, 1.16)	0.89 (0.68, 1.17)	0.90 (0.69, 1.17)	0.89 (0.68, 1.16)	0.88 (0.67, 1.16)	0.86 (0.65, 1.13)	0.92 (0.70, 1.21)	0.88 (0.68, 1.16)	0.89 (0.68, 1.16)	0.88 (0.68, 1.16)	0.92 (0.70, 1.20)	0.88 (0.67, 1.15)
MI	Unexposed	1.00 (., .)	1.00 (., .)	1.00 (., .)	1.00 (., .)	1.00 (., .)	1.00 (., .)	1.00 (., .)	1.00 (., .)	1.00 (., .)	1.00 (., .)	1.00 (., .)	1.00 (., .)
	Tamoxifen	0.47 (0.33, 0.67)	0.47 (0.33, 0.68)	0.46 (0.32, 0.67)	0.47 (0.33, 0.66)	0.45 (0.32, 0.65)	0.46 (0.32, 0.66)	0.47 (0.33, 0.67)	0.47 (0.33, 0.67)	0.47 (0.33, 0.67)	0.47 (0.33, 0.67)	0.47 (0.33, 0.68)	0.47 (0.33, 0.68)
	AI	0.90 (0.74, 1.09)	0.90 (0.75, 1.10)	0.89 (0.74, 1.08)	0.90 (0.74, 1.09)	0.82 (0.67, 1.00)	0.88 (0.72, 1.07)	0.90 (0.74, 1.09)	0.89 (0.74, 1.08)	0.90 (0.74, 1.09)	0.90 (0.74, 1.09)	0.90 (0.74, 1.09)	0.89 (0.73, 1.08)
	Both	0.72 (0.51, 1.02)	0.73 (0.52, 1.03)	0.74 (0.52, 1.04)	0.73 (0.51, 1.03)	0.66 (0.47, 0.94)	0.69 (0.48, 0.98)	0.71 (0.50, 1.00)	0.72 (0.51, 1.01)	0.72 (0.51, 1.02)	0.72 (0.51, 1.02)	0.72 (0.51, 1.02)	0.71 (0.51, 1.01)
Revascularisation	Unexposed	1.00 (., .)	1.00 (., .)	1.00 (., .)	1.00 (., .)	1.00 (., .)	1.00 (., .)	1.00 (., .)	1.00 (., .)	1.00 (., .)	1.00 (., .)	1.00 (., .)	1.00 (., .)
	Tamoxifen	0.65 (0.41, 1.03)	0.65 (0.41, 1.02)	0.62 (0.39, 0.99)	0.62 (0.39, 0.99)	0.65 (0.41, 1.03)	0.68 (0.43, 1.09)	0.65 (0.41, 1.03)	0.65 (0.41, 1.03)	0.65 (0.41, 1.03)	0.64 (0.41, 1.02)	0.65 (0.41, 1.03)	0.65 (0.41, 1.03)
	AI	0.93 (0.70, 1.23)	0.92 (0.70, 1.22)	0.92 (0.70, 1.22)	0.90 (0.68, 1.20)	0.93 (0.70, 1.23)	0.93 (0.70, 1.25)	0.93 (0.70, 1.23)	0.94 (0.71, 1.24)	0.93 (0.70, 1.23)	0.93 (0.71, 1.24)	0.94 (0.71, 1.25)	0.91 (0.69, 1.21)
	Both	0.92 (0.60, 1.43)	0.92 (0.59, 1.42)	0.92 (0.60, 1.43)	0.91 (0.59, 1.41)	0.92 (0.60, 1.43)	0.93 (0.60, 1.46)	0.93 (0.60, 1.44)	0.93 (0.60, 1.44)	0.93 (0.60, 1.43)	0.93 (0.60, 1.43)	0.95 (0.61, 1.47)	0.91 (0.59, 1.41)
SCA	Unexposed	1.00 (., .)	1.00 (., .)	1.00 (., .)	1.00 (., .)	1.00 (., .)	1.00 (., .)	1.00 (., .)	1.00 (., .)	1.00 (., .)	1.00 (., .)	1.00 (., .)	1.00 (., .)
	Tamoxifen	0.72 (0.47, 1.11)	0.72 (0.47, 1.11)	0.71 (0.46, 1.09)	0.70 (0.46, 1.08)	0.68 (0.45, 1.05)	0.71 (0.46, 1.10)	0.72 (0.47, 1.10)	0.72 (0.47, 1.11)	0.72 (0.47, 1.10)	0.72 (0.47, 1.10)	0.72 (0.47, 1.10)	0.72 (0.47, 1.11)
	AI	0.98 (0.75, 1.28)	0.98 (0.75, 1.28)	0.96 (0.74, 1.25)	0.95 (0.73, 1.24)	0.87 (0.66, 1.14)	0.90 (0.68, 1.18)	0.98 (0.75, 1.27)	0.97 (0.75, 1.27)	0.97 (0.75, 1.27)	0.99 (0.76, 1.29)	0.97 (0.74, 1.26)	0.97 (0.74, 1.26)
	Both	0.96 (0.62, 1.48)	0.97 (0.62, 1.49)	0.98 (0.64, 1.52)	0.88 (0.56, 1.37)	0.86 (0.56, 1.34)	0.87 (0.56, 1.37)	0.94 (0.61, 1.45)	0.95 (0.61, 1.47)	0.95 (0.61, 1.47)	0.96 (0.62, 1.48)	0.94 (0.61, 1.45)	0.95 (0.61, 1.46)
PVD	Unexposed	1.00 (., .)	1.00 (., .)	1.00 (., .)	1.00 (., .)	1.00 (., .)	1.00 (., .)	1.00 (., .)	1.00 (., .)	1.00 (., .)	1.00 (., .)	1.00 (., .)	1.00 (., .)
	Tamoxifen	0.88 (0.73, 1.07)	0.88 (0.73, 1.07)	0.89 (0.74, 1.08)	0.88 (0.73, 1.07)	0.87 (0.72, 1.06)	0.89 (0.73, 1.07)	0.89 (0.73, 1.07)	0.88 (0.73, 1.07)	0.88 (0.73, 1.07)	0.88 (0.73, 1.07)	0.88 (0.73, 1.07)	0.88 (0.73, 1.07)
	AI	1.05 (0.92, 1.19)	1.05 (0.92, 1.19)	1.04 (0.92, 1.18)	1.04 (0.92, 1.18)	1.02 (0.90, 1.16)	1.03 (0.90, 1.17)	1.05 (0.93, 1.19)	1.04 (0.92, 1.18)	1.05 (0.92, 1.19)	1.04 (0.92, 1.18)	1.05 (0.93, 1.20)	1.04 (0.91, 1.18)
	Both	0.93 (0.76, 1.15)	0.94 (0.76, 1.15)	0.95 (0.77, 1.16)	0.94 (0.76, 1.15)	0.91 (0.74, 1.12)	0.89 (0.72, 1.10)	0.95 (0.78, 1.17)	0.93 (0.76, 1.14)	0.93 (0.76, 1.15)	0.93 (0.76, 1.14)	0.94 (0.76, 1.15)	0.93 (0.75, 1.14)
Stroke	Unexposed	1.00 (., .)	1.00 (., .)	1.00 (., .)	1.00 (., .)	1.00 (., .)	1.00 (., .)	1.00 (., .)	1.00 (., .)	1.00 (., .)	1.00 (., .)	1.00 (., .)	1.00 (., .)
	Tamoxifen	0.85 (0.72, 1.02)	0.86 (0.72, 1.02)	0.85 (0.71, 1.01)	0.85 (0.71, 1.01)	0.84 (0.71, 1.00)	0.86 (0.72, 1.03)	0.86 (0.72, 1.02)	0.85 (0.72, 1.02)	0.85 (0.72, 1.02)	0.85 (0.72, 1.02)	0.85 (0.72, 1.02)	0.85 (0.72, 1.02)
	AI	0.93 (0.82, 1.04)	0.93 (0.82, 1.04)	0.93 (0.82, 1.04)	0.90 (0.80, 1.01)	0.90 (0.80, 1.01)	0.93 (0.83, 1.05)	0.93 (0.82, 1.04)	0.93 (0.82, 1.04)	0.93 (0.82, 1.04)	0.93 (0.82, 1.04)	0.93 (0.83, 1.05)	0.92 (0.82, 1.04)
	Both	0.84 (0.69, 1.02)	0.84 (0.69, 1.02)	0.84 (0.69, 1.02)	0.83 (0.69, 1.02)	0.82 (0.67, 1.00)	0.84 (0.69, 1.03)	0.84 (0.69, 1.03)	0.84 (0.69, 1.02)	0.84 (0.69, 1.02)	0.84 (0.69, 1.02)	0.85 (0.70, 1.03)	0.84 (0.69, 1.02)
Arrhythmia	Unexposed	1.00 (., .)	1.00 (., .)	1.00 (., .)	1.00 (., .)	1.00 (., .)	1.00 (., .)	1.00 (., .)	1.00 (., .)	1.00 (., .)	1.00 (., .)	1.00 (., .)	1.00 (., .)
	Tamoxifen	0.74 (0.64, 0.87)	0.75 (0.64, 0.87)	0.75 (0.64, 0.87)	0.74 (0.63, 0.87)	0.74 (0.63, 0.86)	0.73 (0.62, 0.86)	0.75 (0.64, 0.88)	0.74 (0.64, 0.87)	0.74 (0.64, 0.87)	0.75 (0.64, 0.87)	0.74 (0.64, 0.87)	0.74 (0.64, 0.87)
	AI	0.95 (0.86, 1.06)	0.95 (0.86, 1.06)	0.95 (0.86, 1.05)	0.95 (0.85, 1.05)	0.93 (0.84, 1.03)	0.94 (0.85, 1.05)	0.96 (0.86, 1.06)	0.95 (0.86, 1.05)	0.95 (0.86, 1.05)	0.95 (0.86, 1.05)	0.95 (0.86, 1.06)	0.95 (0.86, 1.05)
	Both	0.86 (0.73, 1.02)	0.87 (0.73, 1.03)	0.87 (0.73, 1.03)	0.85 (0.72, 1.01)	0.84 (0.71, 1.00)	0.86 (0.72, 1.02)	0.87 (0.73, 1.03)	0.86 (0.73, 1.02)	0.86 (0.73, 1.02)	0.86 (0.73, 1.02)	0.86 (0.73, 1.02)	0.86 (0.72, 1.01)
HF	Unexposed	1.00 (., .)	1.00 (., .)	1.00 (., .)	1.00 (., .)	1.00 (., .)	1.00 (., .)	1.00 (., .)	1.00 (., .)	1.00 (., .)	1.00 (., .)	1.00 (., .)	1.00 (., .)
	Tamoxifen	0.86 (0.74, 1.01)	0.87 (0.74, 1.01)	0.87 (0.74, 1.01)	0.86 (0.74, 1.01)	0.84 (0.72, 0.98)	0.85 (0.73, 1.00)	0.87 (0.74, 1.02)	0.86 (0.74, 1.01)	0.86 (0.74, 1.01)	0.87 (0.74, 1.01)	0.86 (0.74, 1.01)	0.86 (0.74, 1.01)
	AI	0.93 (0.84, 1.03)	0.93 (0.84, 1.04)	0.93 (0.83, 1.03)	0.90 (0.81, 1.01)	0.87 (0.78, 0.97)	0.91 (0.82, 1.02)	0.93 (0.84, 1.04)	0.92 (0.83, 1.03)	0.92 (0.83, 1.03)	0.92 (0.83, 1.03)	0.93 (0.84, 1.04)	0.92 (0.82, 1.02)
	Both	0.86 (0.72, 1.03)	0.86 (0.72, 1.03)	0.87 (0.73, 1.04)	0.85 (0.71, 1.02)	0.81 (0.68, 0.97)	0.83 (0.69, 1.00)	0.88 (0.73, 1.05)	0.85 (0.71, 1.02)	0.86 (0.72, 1.02)	0.85 (0.71, 1.02)	0.86 (0.72, 1.03)	0.85 (0.71, 1.02)
Pericarditis	Unexposed	1.00 (., .)	1.00 (., .)	1.00 (., .)	1.00 (., .)	1.00 (., .)	1.00 (., .)	1.00 (., .)	1.00 (., .)	1.00 (., .)	1.00 (., .)	1.00 (., .)	1.00 (., .)
	Tamoxifen	0.39 (0.23, 0.67)	0.39 (0.22, 0.66)	0.39 (0.23, 0.67)	0.38 (0.22, 0.65)	0.38 (0.22, 0.65)	0.37 (0.21, 0.64)	0.39 (0.23, 0.67)	0.39 (0.23, 0.66)	0.39 (0.22, 0.66)	0.39 (0.23, 0.67)	0.39 (0.23, 0.67)	0.39 (0.22, 0.66)
	AI	0.78 (0.59, 1.02)	0.77 (0.59, 1.02)	0.78 (0.59, 1.03)	0.76 (0.57, 1.00)	0.72 (0.54, 0.95)	0.72 (0.54, 0.95)	0.78 (0.60, 1.03)	0.74 (0.56, 0.97)	0.76 (0.58, 1.01)	0.77 (0.58, 1.02)	0.77 (0.58, 1.01)	0.75 (0.57, 0.98)
	Both	0.61 (0.37, 1.01)	0.61 (0.37, 1.00)	0.63 (0.38, 1.04)	0.61 (0.37, 1.00)	0.58 (0.35, 0.95)	0.54 (0.32, 0.91)	0.64 (0.39, 1.05)	0.59 (0.36, 0.97)	0.61 (0.37, 1.00)	0.61 (0.37, 1.01)	0.60 (0.37, 0.99)	0.60 (0.36, 0.98)
VHD	Unexposed	1.00 (., .)	1.00 (., .)	1.00 (., .)	1.00 (., .)	1.00 (., .)	1.00 (., .)	1.00 (., .)	1.00 (., .)	1.00 (., .)	1.00 (., .)	1.00 (., .)	1.00 (., .)
	Tamoxifen	0.78 (0.66, 0.92)	0.79 (0.67, 0.93)	0.78 (0.66, 0.92)	0.77 (0.65, 0.91)	0.78 (0.66, 0.92)	0.79 (0.67, 0.93)	0.79 (0.67, 0.94)	0.78 (0.66, 0.92)	0.78 (0.66, 0.92)	0.79 (0.67, 0.93)	0.78 (0.66, 0.92)	0.78 (0.66, 0.92)
	AI	1.02 (0.92, 1.14)	1.03 (0.92, 1.14)	1.02 (0.92, 1.14)	0.99 (0.89, 1.10)	1.01 (0.90, 1.13)	1.00 (0.89, 1.12)	1.03 (0.93, 1.15)	1.02 (0.91, 1.13)	1.02 (0.92, 1.14)	1.02 (0.91, 1.13)	1.03 (0.93, 1.15)	1.01 (0.91, 1.13)
	Both	0.92 (0.77, 1.09)	0.93 (0.78, 1.11)	0.93 (0.78, 1.11)	0.90 (0.76, 1.08)	0.91 (0.76, 1.08)	0.88 (0.74, 1.06)	0.96 (0.81, 1.15)	0.91 (0.76, 1.09)	0.91 (0.77, 1.09)	0.91 (0.77, 1.09)	0.93 (0.78, 1.11)	0.91 (0.76, 1.08)
VTE	Unexposed	1.00 (., .)	1.00 (., .)	1.00 (., .)	1.00 (., .)	1.00 (., .)	1.00 (., .)	1.00 (., .)	1.00 (., .)	1.00 (., .)	1.00 (., .)	1.00 (., .)	1.00 (., .)
	Tamoxifen	1.35 (0.96, 1.90)	1.35 (0.96, 1.90)	1.34 (0.95, 1.89)	1.33 (0.95, 1.88)	1.31 (0.93, 1.84)	1.41 (1.00, 2.00)	1.36 (0.97, 1.91)	1.35 (0.96, 1.90)	1.34 (0.96, 1.89)	1.35 (0.96, 1.90)	1.35 (0.96, 1.90)	1.35 (0.96, 1.90)
	AI	1.15 (0.89, 1.48)	1.15 (0.89, 1.49)	1.15 (0.89, 1.48)	1.12 (0.87, 1.45)	1.06 (0.82, 1.38)	1.16 (0.89, 1.52)	1.16 (0.89, 1.49)	1.12 (0.87, 1.45)	1.13 (0.88, 1.46)	1.15 (0.89, 1.48)	1.17 (0.91, 1.51)	1.12 (0.87, 1.45)
	Both	1.71 (1.20, 2.42)	1.71 (1.21, 2.44)	1.76 (1.24, 2.50)	1.68 (1.18, 2.39)	1.59 (1.12, 2.27)	1.68 (1.17, 2.42)	1.76 (1.23, 2.50)	1.68 (1.18, 2.39)	1.69 (1.19, 2.40)	1.71 (1.20, 2.42)	1.75 (1.23, 2.49)	1.68 (1.18, 2.39)
DVT	Unexposed	1.00 (., .)	1.00 (., .)	1.00 (., .)	1.00 (., .)	1.00 (., .)	1.00 (., .)	1.00 (., .)	1.00 (., .)	1.00 (., .)	1.00 (., .)	1.00 (., .)	1.00 (., .)
	Tamoxifen	1.36 (0.96, 1.94)	1.37 (0.96, 1.95)	1.35 (0.95, 1.93)	1.35 (0.95, 1.93)	1.32 (0.93, 1.89)	1.44 (1.00, 2.07)	1.38 (0.97, 1.96)	1.36 (0.96, 1.94)	1.36 (0.95, 1.94)	1.36 (0.96, 1.94)	1.36 (0.96, 1.94)	1.36 (0.96, 1.94)
	AI	1.15 (0.88, 1.51)	1.16 (0.89, 1.51)	1.15 (0.88, 1.50)	1.13 (0.86, 1.47)	1.07 (0.82, 1.40)	1.17 (0.89, 1.55)	1.16 (0.89, 1.52)	1.13 (0.86, 1.48)	1.14 (0.87, 1.49)	1.15 (0.88, 1.51)	1.18 (0.90, 1.54)	1.13 (0.87, 1.48)
	Both	1.82 (1.27, 2.60)	1.83 (1.28, 2.62)	1.87 (1.30, 2.67)	1.79 (1.25, 2.57)	1.70 (1.19, 2.45)	1.80 (1.24, 2.61)	1.88 (1.31, 2.70)	1.79 (1.25, 2.56)	1.80 (1.26, 2.58)	1.82 (1.27, 2.60)	1.88 (1.31, 2.69)	1.80 (1.25, 2.57)
PE	Unexposed	1.00 (., .)	1.00 (., .)	1.00 (., .)	1.00 (., .)	1.00 (., .)	1.00 (., .)	1.00 (., .)	1.00 (., .)	1.00 (., .)	1.00 (., .)	1.00 (., .)	1.00 (., .)
	Tamoxifen	1.03 (0.34, 3.09)	1.04 (0.35, 3.10)	1.06 (0.35, 3.17)	1.00 (0.33, 3.00)	0.98 (0.33, 2.93)	1.02 (0.34, 3.05)	1.03 (0.34, 3.09)	1.03 (0.34, 3.10)	1.02 (0.34, 3.07)	1.03 (0.35, 3.10)	1.04 (0.35, 3.11)	1.03 (0.34, 3.09)
	AI	1.04 (0.48, 2.24)	1.03 (0.48, 2.24)	1.01 (0.47, 2.19)	0.96 (0.44, 2.07)	0.92 (0.42, 2.01)	0.99 (0.46, 2.16)	1.03 (0.48, 2.23)	1.01 (0.46, 2.18)	1.01 (0.47, 2.19)	1.03 (0.48, 2.23)	1.02 (0.47, 2.21)	0.99 (0.46, 2.15)
	Both	0.28 (0.03, 2.21)	0.28 (0.03, 2.20)	0.29 (0.04, 2.27)	0.27 (0.03, 2.14)	0.25 (0.03, 1.98)	0.27 (0.03, 2.16)	0.27 (0.03, 2.17)	0.27 (0.03, 2.16)	0.27 (0.03, 2.16)	0.28 (0.03, 2.20)	0.27 (0.03, 2.17)	0.27 (0.03, 2.14)

Matthews AA, *et al. Heart* 2021; 107:1327–1335. doi: 10.1136/heartjnl-2020-317510

Appendix 19B – Sensitivity analysis - Crude and individually adjusted association between ever exposure to endocrine therapies and all CVD outcomes in the US, continued

Outcome	Exposure	Crude (with age as a timescale)	RA	CKD	Hypertension	Diabetes	Statins	Hyptertensive drugs	ACE inhibitors	CCBs	ARBs	Prior non-Venous CVD	Prior VTE
Coronary Artery Disease	Unexposed	1.00 (., .)	1.00 (., .)	1.00 (., .)	1.00 (., .)	1.00 (., .)	1.00 (., .)	1.00 (., .)	1.00 (., .)	1.00 (., .)	1.00 (., .)	1.00 (., .)	1.00 (., .)
	Tamoxifen	0.73 (0.60, 0.90)	0.73 (0.60, 0.90)	0.74 (0.60, 0.91)	0.74 (0.60, 0.91)	0.73 (0.60, 0.90)	0.73 (0.60, 0.90)	0.73 (0.59, 0.90)	0.73 (0.60, 0.90)	0.74 (0.60, 0.91)	0.73 (0.60, 0.90)	0.75 (0.61, 0.93)	0.74 (0.60, 0.91)
	AI	1.04 (0.91, 1.18)	1.04 (0.92, 1.19)	1.04 (0.91, 1.18)	1.01 (0.89, 1.15)	1.02 (0.90, 1.16)	1.03 (0.91, 1.17)	1.03 (0.91, 1.18)	1.04 (0.91, 1.18)	1.04 (0.91, 1.18)	1.03 (0.91, 1.18)	1.02 (0.90, 1.16)	1.04 (0.91, 1.18)
	Both	0.89 (0.72, 1.11)	0.90 (0.72, 1.11)	0.90 (0.73, 1.12)	0.89 (0.72, 1.10)	0.90 (0.73, 1.11)	0.89 (0.72, 1.10)	0.89 (0.72, 1.10)	0.90 (0.72, 1.11)	0.89 (0.72, 1.11)	0.89 (0.72, 1.10)	0.90 (0.73, 1.12)	0.90 (0.72, 1.11)
Angina	Unexposed	1.00 (., .)	1.00 (., .)	1.00 (., .)	1.00 (., .)	1.00 (., .)	1.00 (., .)	1.00 (., .)	1.00 (., .)	1.00 (., .)	1.00 (., .)	1.00 (., .)	1.00 (., .)
	Tamoxifen	0.82 (0.63, 1.05)	0.81 (0.63, 1.05)	0.82 (0.64, 1.06)	0.83 (0.64, 1.07)	0.81 (0.63, 1.05)	0.82 (0.63, 1.05)	0.81 (0.63, 1.05)	0.82 (0.63, 1.05)	0.82 (0.64, 1.06)	0.82 (0.63, 1.06)	0.84 (0.65, 1.08)	0.82 (0.64, 1.06)
	AI	1.08 (0.92, 1.27)	1.09 (0.92, 1.28)	1.08 (0.92, 1.27)	1.05 (0.89, 1.24)	1.06 (0.90, 1.25)	1.07 (0.91, 1.26)	1.08 (0.91, 1.27)	1.08 (0.91, 1.27)	1.08 (0.91, 1.27)	1.07 (0.91, 1.26)	1.06 (0.90, 1.25)	1.08 (0.92, 1.27)
	Both	0.88 (0.68, 1.16)	0.89 (0.68, 1.16)	0.89 (0.68, 1.17)	0.88 (0.67, 1.15)	0.89 (0.68, 1.17)	0.88 (0.67, 1.15)	0.88 (0.67, 1.15)	0.89 (0.68, 1.16)	0.89 (0.68, 1.16)	0.88 (0.67, 1.15)	0.89 (0.68, 1.17)	0.89 (0.68, 1.16)
MI	Unexposed	1.00 (., .)	1.00 (., .)	1.00 (., .)	1.00 (., .)	1.00 (., .)	1.00 (., .)	1.00 (., .)	1.00 (., .)	1.00 (., .)	1.00 (., .)	1.00 (., .)	1.00 (., .)
	Tamoxifen	0.47 (0.33, 0.67)	0.47 (0.33, 0.67)	0.47 (0.33, 0.68)	0.48 (0.33, 0.68)	0.48 (0.33, 0.68)	0.47 (0.33, 0.67)	0.47 (0.33, 0.67)	0.47 (0.33, 0.67)	0.47 (0.33, 0.67)	0.47 (0.33, 0.67)	0.49 (0.34, 0.69)	0.48 (0.33, 0.68)
	AI	0.90 (0.74, 1.09)	0.91 (0.75, 1.10)	0.90 (0.74, 1.09)	0.89 (0.73, 1.07)	0.88 (0.73, 1.07)	0.89 (0.74, 1.08)	0.90 (0.74, 1.09)	0.90 (0.74, 1.09)	0.90 (0.74, 1.09)	0.90 (0.74, 1.09)	0.89 (0.74, 1.08)	0.90 (0.75, 1.10)
	Both	0.72 (0.51, 1.02)	0.72 (0.51, 1.02)	0.73 (0.52, 1.04)	0.72 (0.51, 1.02)	0.73 (0.52, 1.04)	0.72 (0.51, 1.02)	0.72 (0.51, 1.02)	0.73 (0.51, 1.03)	0.72 (0.51, 1.02)	0.72 (0.51, 1.02)	0.74 (0.52, 1.04)	0.73 (0.51, 1.03)
Revascularisation	Unexposed	1.00 (., .)	1.00 (., .)	1.00 (., .)	1.00 (., .)	1.00 (., .)	1.00 (., .)	1.00 (., .)	1.00 (., .)	1.00 (., .)	1.00 (., .)	1.00 (., .)	1.00 (., .)
	Tamoxifen	0.65 (0.41, 1.03)	0.64 (0.40, 1.02)	0.65 (0.41, 1.03)	0.65 (0.41, 1.04)	0.65 (0.41, 1.03)	0.65 (0.41, 1.03)	0.65 (0.41, 1.03)	0.64 (0.41, 1.02)	0.65 (0.41, 1.03)	0.65 (0.41, 1.03)	0.66 (0.42, 1.05)	0.65 (0.41, 1.03)
	AI	0.93 (0.70, 1.23)	0.93 (0.70, 1.23)	0.92 (0.70, 1.22)	0.90 (0.68, 1.19)	0.90 (0.68, 1.19)	0.93 (0.70, 1.23)	0.92 (0.70, 1.22)	0.92 (0.70, 1.22)	0.93 (0.70, 1.23)	0.92 (0.70, 1.22)	0.92 (0.70, 1.22)	0.93 (0.70, 1.23)
	Both	0.92 (0.60, 1.43)	0.93 (0.60, 1.43)	0.94 (0.61, 1.45)	0.92 (0.59, 1.42)	0.94 (0.61, 1.46)	0.92 (0.60, 1.43)	0.92 (0.60, 1.42)	0.93 (0.60, 1.43)	0.92 (0.60, 1.43)	0.92 (0.59, 1.42)	0.94 (0.61, 1.46)	0.93 (0.60, 1.43)
SCA	Unexposed	1.00 (., .)	1.00 (., .)	1.00 (., .)	1.00 (., .)	1.00 (., .)	1.00 (., .)	1.00 (., .)	1.00 (., .)	1.00 (., .)	1.00 (., .)	1.00 (., .)	1.00 (., .)
	Tamoxifen	0.72 (0.47, 1.11)	0.72 (0.47, 1.10)	0.72 (0.47, 1.11)	0.73 (0.48, 1.12)	0.73 (0.48, 1.12)	0.72 (0.47, 1.11)	0.71 (0.46, 1.09)	0.72 (0.47, 1.10)	0.72 (0.47, 1.11)	0.72 (0.47, 1.11)	0.74 (0.48, 1.14)	0.73 (0.48, 1.12)
	AI	0.98 (0.75, 1.28)	0.98 (0.76, 1.28)	0.98 (0.75, 1.27)	0.96 (0.74, 1.25)	0.97 (0.74, 1.26)	0.98 (0.75, 1.28)	0.97 (0.75, 1.27)	0.98 (0.75, 1.27)	0.98 (0.75, 1.28)	0.98 (0.75, 1.27)	0.97 (0.74, 1.26)	0.98 (0.75, 1.28)
	Both	0.96 (0.62, 1.48)	0.96 (0.62, 1.48)	0.99 (0.64, 1.53)	0.96 (0.62, 1.48)	0.97 (0.63, 1.50)	0.96 (0.62, 1.48)	0.95 (0.61, 1.46)	0.96 (0.62, 1.48)	0.96 (0.62, 1.48)	0.95 (0.62, 1.48)	0.97 (0.63, 1.50)	0.96 (0.62, 1.48)
PVD	Unexposed	1.00 (., .)	1.00 (., .)	1.00 (., .)	1.00 (., .)	1.00 (., .)	1.00 (., .)	1.00 (., .)	1.00 (., .)	1.00 (., .)	1.00 (., .)	1.00 (., .)	1.00 (., .)
	Tamoxifen	0.88 (0.73, 1.07)	0.88 (0.73, 1.06)	0.89 (0.73, 1.07)	0.89 (0.73, 1.07)	0.89 (0.73, 1.07)	0.88 (0.73, 1.07)	0.88 (0.73, 1.06)	0.88 (0.73, 1.06)	0.88 (0.73, 1.07)	0.88 (0.73, 1.07)	0.91 (0.75, 1.09)	0.89 (0.73, 1.07)
	AI	1.05 (0.92, 1.19)	1.05 (0.92, 1.19)	1.05 (0.92, 1.19)	1.02 (0.90, 1.16)	1.03 (0.91, 1.17)	1.04 (0.92, 1.18)	1.04 (0.92, 1.19)	1.04 (0.92, 1.19)	1.04 (0.92, 1.19)	1.04 (0.92, 1.18)	1.04 (0.91, 1.18)	1.05 (0.92, 1.19)
	Both	0.93 (0.76, 1.15)	0.93 (0.76, 1.14)	0.95 (0.77, 1.17)	0.93 (0.76, 1.14)	0.95 (0.77, 1.16)	0.93 (0.76, 1.14)	0.93 (0.76, 1.14)	0.94 (0.76, 1.15)	0.93 (0.76, 1.14)	0.93 (0.76, 1.14)	0.94 (0.77, 1.15)	0.94 (0.76, 1.15)
Stroke	Unexposed	1.00 (., .)	1.00 (., .)	1.00 (., .)	1.00 (., .)	1.00 (., .)	1.00 (., .)	1.00 (., .)	1.00 (., .)	1.00 (., .)	1.00 (., .)	1.00 (., .)	1.00 (., .)
	Tamoxifen	0.85 (0.72, 1.02)	0.85 (0.72, 1.02)	0.86 (0.72, 1.02)	0.86 (0.72, 1.02)	0.85 (0.72, 1.02)	0.85 (0.72, 1.01)	0.85 (0.72, 1.01)	0.85 (0.72, 1.01)	0.85 (0.72, 1.02)	0.86 (0.72, 1.02)	0.86 (0.73, 1.03)	0.86 (0.72, 1.02)
	AI	0.93 (0.82, 1.04)	0.93 (0.83, 1.04)	0.92 (0.82, 1.04)	0.91 (0.81, 1.03)	0.91 (0.81, 1.02)	0.92 (0.81, 1.03)	0.92 (0.82, 1.04)	0.92 (0.82, 1.04)	0.92 (0.82, 1.04)	0.93 (0.82, 1.04)	0.92 (0.81, 1.03)	0.93 (0.82, 1.04)
	Both	0.84 (0.69, 1.02)	0.84 (0.69, 1.02)	0.85 (0.69, 1.03)	0.84 (0.69, 1.02)	0.84 (0.69, 1.03)	0.84 (0.69, 1.02)	0.84 (0.69, 1.02)	0.84 (0.69, 1.02)	0.84 (0.69, 1.02)	0.84 (0.69, 1.02)	0.85 (0.70, 1.03)	0.84 (0.69, 1.03)
Arrhythmia	Unexposed	1.00 (., .)	1.00 (., .)	1.00 (., .)	1.00 (., .)	1.00 (., .)	1.00 (., .)	1.00 (., .)	1.00 (., .)	1.00 (., .)	1.00 (., .)	1.00 (., .)	1.00 (., .)
	Tamoxifen	0.74 (0.64, 0.87)	0.74 (0.63, 0.87)	0.75 (0.64, 0.88)	0.75 (0.64, 0.87)	0.75 (0.64, 0.88)	0.74 (0.64, 0.87)	0.74 (0.63, 0.87)	0.74 (0.63, 0.87)	0.75 (0.64, 0.88)	0.75 (0.64, 0.87)	0.76 (0.65, 0.89)	0.75 (0.64, 0.88)
	AI	0.95 (0.86, 1.06)	0.95 (0.86, 1.06)	0.95 (0.86, 1.06)	0.94 (0.85, 1.04)	0.94 (0.85, 1.04)	0.95 (0.85, 1.05)	0.95 (0.86, 1.05)	0.95 (0.86, 1.05)	0.95 (0.86, 1.06)	0.95 (0.86, 1.05)	0.94 (0.85, 1.04)	0.95 (0.86, 1.06)
	Both	0.86 (0.73, 1.02)	0.86 (0.73, 1.02)	0.87 (0.73, 1.03)	0.86 (0.73, 1.02)	0.87 (0.73, 1.03)	0.86 (0.73, 1.02)	0.86 (0.73, 1.02)	0.86 (0.73, 1.02)	0.86 (0.73, 1.02)	0.86 (0.73, 1.02)	0.88 (0.74, 1.04)	0.86 (0.73, 1.02)
HF	Unexposed	1.00 (., .)	1.00 (., .)	1.00 (., .)	1.00 (., .)	1.00 (., .)	1.00 (., .)	1.00 (., .)	1.00 (., .)	1.00 (., .)	1.00 (., .)	1.00 (., .)	1.00 (., .)
	Tamoxifen	0.86 (0.74, 1.01)	0.86 (0.74, 1.01)	0.87 (0.74, 1.01)	0.87 (0.74, 1.02)	0.87 (0.74, 1.02)	0.86 (0.74, 1.01)	0.86 (0.73, 1.00)	0.86 (0.74, 1.01)	0.86 (0.74, 1.01)	0.87 (0.74, 1.01)	0.88 (0.76, 1.03)	0.88 (0.75, 1.02)
	AI	0.93 (0.84, 1.03)	0.93 (0.84, 1.04)	0.93 (0.84, 1.03)	0.91 (0.82, 1.01)	0.91 (0.82, 1.02)	0.92 (0.82, 1.02)	0.93 (0.83, 1.03)	0.92 (0.83, 1.03)	0.93 (0.83, 1.03)	0.93 (0.83, 1.03)	0.92 (0.83, 1.02)	0.93 (0.84, 1.03)
	Both	0.86 (0.72, 1.03)	0.86 (0.72, 1.03)	0.87 (0.73, 1.04)	0.85 (0.72, 1.02)	0.87 (0.73, 1.04)	0.85 (0.72, 1.02)	0.86 (0.72, 1.03)	0.87 (0.72, 1.03)	0.86 (0.72, 1.03)	0.86 (0.72, 1.02)	0.87 (0.72, 1.03)	0.86 (0.72, 1.03)
Pericarditis	Unexposed	1.00 (., .)	1.00 (., .)	1.00 (., .)	1.00 (., .)	1.00 (., .)	1.00 (., .)	1.00 (., .)	1.00 (., .)	1.00 (., .)	1.00 (., .)	1.00 (., .)	1.00 (., .)
	Tamoxifen	0.39 (0.23, 0.67)	0.39 (0.23, 0.67)	0.39 (0.23, 0.67)	0.39 (0.23, 0.67)	0.39 (0.23, 0.67)	0.39 (0.23, 0.67)	0.39 (0.22, 0.66)	0.39 (0.23, 0.66)	0.39 (0.23, 0.67)	0.39 (0.23, 0.66)	0.40 (0.23, 0.69)	0.39 (0.23, 0.67)
	AI	0.78 (0.59, 1.02)	0.78 (0.59, 1.03)	0.78 (0.59, 1.02)	0.76 (0.58, 1.01)	0.77 (0.59, 1.01)	0.78 (0.59, 1.02)	0.77 (0.59, 1.02)	0.78 (0.59, 1.02)	0.78 (0.59, 1.02)	0.78 (0.59, 1.03)	0.77 (0.59, 1.01)	0.78 (0.59, 1.02)
	Both	0.61 (0.37, 1.01)	0.62 (0.37, 1.01)	0.63 (0.38, 1.03)	0.61 (0.37, 1.01)	0.62 (0.38, 1.02)	0.61 (0.37, 1.01)	0.61 (0.37, 1.01)	0.62 (0.37, 1.01)	0.62 (0.37, 1.01)	0.61 (0.37, 1.01)	0.63 (0.38, 1.03)	0.61 (0.37, 1.01)
VHD	Unexposed	1.00 (., .)	1.00 (., .)	1.00 (., .)	1.00 (., .)	1.00 (., .)	1.00 (., .)	1.00 (., .)	1.00 (., .)	1.00 (., .)	1.00 (., .)	1.00 (., .)	1.00 (., .)
	Tamoxifen	0.78 (0.66, 0.92)	0.78 (0.66, 0.92)	0.78 (0.66, 0.93)	0.79 (0.67, 0.93)	0.79 (0.67, 0.93)	0.78 (0.66, 0.92)	0.78 (0.66, 0.92)	0.78 (0.66, 0.92)	0.79 (0.67, 0.93)	0.78 (0.66, 0.93)	0.80 (0.68, 0.94)	0.79 (0.67, 0.93)
	AI	1.02 (0.92, 1.14)	1.03 (0.92, 1.14)	1.02 (0.92, 1.14)	1.01 (0.91, 1.12)	1.02 (0.91, 1.13)	1.01 (0.91, 1.13)	1.02 (0.92, 1.14)	1.02 (0.92, 1.14)	1.02 (0.92, 1.14)	1.02 (0.91, 1.13)	1.02 (0.91, 1.13)	1.02 (0.92, 1.14)
	Both	0.92 (0.77, 1.09)	0.92 (0.77, 1.10)	0.92 (0.77, 1.10)	0.92 (0.77, 1.10)	0.92 (0.77, 1.10)	0.91 (0.77, 1.09)	0.91 (0.77, 1.09)	0.92 (0.77, 1.10)	0.92 (0.77, 1.10)	0.91 (0.76, 1.09)	0.93 (0.78, 1.11)	0.92 (0.77, 1.10)
VTE	Unexposed	1.00 (., .)	1.00 (., .)	1.00 (., .)	1.00 (., .)	1.00 (., .)	1.00 (., .)	1.00 (., .)	1.00 (., .)	1.00 (., .)	1.00 (., .)	1.00 (., .)	1.00 (., .)
	Tamoxifen	1.35 (0.96, 1.90)	1.35 (0.96, 1.90)	1.35 (0.96, 1.90)	1.36 (0.97, 1.91)	1.35 (0.96, 1.90)	1.35 (0.96, 1.90)	1.35 (0.96, 1.89)	1.34 (0.96, 1.89)	1.35 (0.96, 1.90)	1.35 (0.96, 1.90)	1.37 (0.98, 1.93)	1.35 (0.96, 1.90)
	AI	1.15 (0.89, 1.48)	1.15 (0.89, 1.49)	1.15 (0.89, 1.48)	1.13 (0.88, 1.47)	1.14 (0.88, 1.47)	1.15 (0.89, 1.48)	1.15 (0.89, 1.48)	1.14 (0.89, 1.48)	1.15 (0.89, 1.48)	1.15 (0.89, 1.49)	1.14 (0.88, 1.47)	1.15 (0.89, 1.48)
	Both	1.71 (1.20, 2.42)	1.71 (1.20, 2.43)	1.71 (1.21, 2.43)	1.71 (1.20, 2.43)	1.72 (1.21, 2.44)	1.71 (1.20, 2.42)	1.70 (1.20, 2.42)	1.71 (1.21, 2.43)	1.71 (1.20, 2.43)	1.71 (1.20, 2.43)	1.73 (1.21, 2.45)	1.71 (1.20, 2.42)
DVT	Unexposed	1.00 (., .)	1.00 (., .)	1.00 (., .)	1.00 (., .)	1.00 (., .)	1.00 (., .)	1.00 (., .)	1.00 (., .)	1.00 (., .)	1.00 (., .)	1.00 (., .)	1.00 (., .)
	Tamoxifen	1.36 (0.96, 1.94)	1.36 (0.96, 1.94)	1.36 (0.96, 1.94)	1.37 (0.96, 1.95)	1.37 (0.96, 1.95)	1.36 (0.96, 1.94)	1.36 (0.96, 1.94)	1.36 (0.95, 1.94)	1.36 (0.96, 1.94)	1.36 (0.96, 1.94)	1.39 (0.97, 1.97)	1.37 (0.96, 1.95)
	AI	1.15 (0.88, 1.51)	1.15 (0.88, 1.51)	1.15 (0.88, 1.50)	1.14 (0.87, 1.49)	1.14 (0.88, 1.49)	1.16 (0.89, 1.51)	1.15 (0.88, 1.51)	1.15 (0.88, 1.50)	1.15 (0.88, 1.51)	1.15 (0.88, 1.51)	1.14 (0.88, 1.49)	1.16 (0.89, 1.51)
	Both	1.82 (1.27, 2.60)	1.82 (1.27, 2.60)	1.82 (1.27, 2.61)	1.82 (1.27, 2.60)	1.83 (1.28, 2.62)	1.82 (1.27, 2.60)	1.82 (1.27, 2.60)	1.82 (1.27, 2.61)	1.82 (1.27, 2.60)	1.82 (1.27, 2.60)	1.83 (1.28, 2.63)	1.82 (1.27, 2.61)
PE	Unexposed	1.00 (., .)	1.00 (., .)	1.00 (., .)	1.00 (., .)	1.00 (., .)	1.00 (., .)	1.00 (., .)	1.00 (., .)	1.00 (., .)	1.00 (., .)	1.00 (., .)	1.00 (., .)
	Tamoxifen	1.03 (0.34, 3.09)	1.03 (0.34, 3.10)	1.03 (0.34, 3.09)	1.05 (0.35, 3.14)	1.04 (0.35, 3.11)	1.03 (0.34, 3.09)	1.02 (0.34, 3.05)	1.03 (0.34, 3.09)	1.04 (0.35, 3.11)	1.03 (0.34, 3.10)	1.07 (0.36, 3.21)	1.05 (0.35, 3.15)
	AI	1.04 (0.48, 2.24)	1.05 (0.48, 2.26)	1.04 (0.48, 2.24)	1.00 (0.46, 2.17)	1.03 (0.48, 2.23)	1.03 (0.47, 2.22)	1.02 (0.47, 2.20)	1.04 (0.48, 2.24)	1.04 (0.48, 2.24)	1.04 (0.48, 2.24)	1.02 (0.47, 2.21)	1.04 (0.48, 2.24)
	Both	0.28 (0.03, 2.21)	0.28 (0.04, 2.22)	0.28 (0.04, 2.22)	0.28 (0.03, 2.22)	0.28 (0.04, 2.22)	0.28 (0.03, 2.20)	0.27 (0.03, 2.19)	0.28 (0.03, 2.21)	0.28 (0.03, 2.22)	0.28 (0.03, 2.21)	0.28 (0.04, 2.24)	0.28 (0.03, 2.20)

Matthews AA, *et al. Heart* 2021; 107:1327–1335. doi: 10.1136/heartjnl-2020-317510

REFERENCES

1. Herrett, E., et al., *Data Resource Profile: Clinical Practice Research Datalink (CPRD).* Int J Epidemiol, 2015. **44**(3): p. 827-36.
2. Campbell, J.D., D.J. Eaton, S.C. Gallagher, A.M. Williams, T.J., *Is the CPRD GOLD Population Comparable to the U.K. Population?* Pharmacoepidemiology and Drug Safety, 2013. **22**(Suppl 1): p. 280.
3. *Hospital Episode Statistics*. 2016 11/12/2017].
4. National Cancer Institute. *Overview of the SEER program*. 30/8/2018].
5. Nattinger, A.B., T.L. McAuliffe, and M.M. Schapira, *Generalizability of the surveillance, epidemiology, and end results registry population: factors relevant to epidemiologic and health care research.* J Clin Epidemiol, 1997. **50**(8): p. 939-45.
6. Warren, J.L., et al., *Overview of the SEER-Medicare data: content, research applications, and generalizability to the United States elderly population.* Med Care, 2002. **40**(8 Suppl): p. IV-3-18.
7. Engels, E.A., et al., *Use of surveillance, epidemiology, and end results-medicare data to conduct case-control studies of cancer among the US elderly.* Am J Epidemiol, 2011. **174**(7): p. 860-70.
8. Levey, A.S., et al., *A new equation to estimate glomerular filtration rate.* Ann Intern Med, 2009. **150**(9): p. 604-12.
9. Fox, C.S., et al., *Associations of kidney disease measures with mortality and end-stage renal disease in individuals with and without diabetes: a meta-analysis.* Lancet, 2012. **380**(9854): p. 1662-73.
10. Bhaskaran, K., et al., *Body-mass index and risk of 22 specific cancers: a population-based cohort study of 5.24 million UK adults.* Lancet, 2014. **384**(9945): p. 755-65.
11. Noble, M.M., et al., *The English Indices of Deprivation 2010.* D.f.C.a.L. Government, 2011.
12. Lash, T.F., MP. Fink, AK., *Applying Quantitative Bias Analysis to Epidemiologic Data*. 2009: Springer.
13. Sheppard, V.B., et al., *Frailty and adherence to adjuvant hormonal therapy in older women with breast cancer: CALGB protocol 369901.* J Clin Oncol, 2014. **32**(22): p. 2318-27.
14. Veronese, N., et al., *Frailty and Risk of Cardiovascular Diseases in Older Persons: The Age, Gene/Environment Susceptibility-Reykjavik Study.* Rejuvenation Res, 2017. **20**(6): p. 517-524.
